# Tumor‐Associated Glycan Exploits Adenosine Receptor 2A Signaling to Facilitate Immune Evasion

**DOI:** 10.1002/advs.202416501

**Published:** 2025-06-18

**Authors:** Jing‐Yan Cheng, Hsiu‐Hui Tsai, Jung‐Tung Hung, Tsai‐Hsien Hung, Chun‐Cheng Lin, Chien‐Wei Lee, Zi‐Chi Lo, Jing‐Rong Huang, Shih‐Pin Chiou, Yenlin Huang, Shih‐Hsiang Chen, Chun‐Nan Yeh, John Yu, Alice L. Yu

**Affiliations:** ^1^ Institute of Stem Cell and Translational Cancer Research Chang Gung Memorial Hospital Linkou Medical Center 15, Wenhua 1st Rd. Taoyuan 333 Taiwan; ^2^ Department of Chemistry National Tsing Hua University 101, Sec. 2, Kuang‐Fu Rd. Hsinchu 300 Taiwan; ^3^ Department of Anatomic Pathology Chang Gung Memorial Hospital at Linko 5, Fuxing St. Taoyuan 333 Taiwan; ^4^ School of Medicine National Tsing Hua University 101, Sec. 2, Kuang‐Fu Rd. Hsinchu 300 Taiwan; ^5^ Department of Pediatrics Division of Hematology‐Oncology Chang Gung Memorial Hospital 5, Fuxing St. Taoyuan 333 Taiwan; ^6^ College of Medicine Chang Gung University 259, Wenhua 1st Rd. Taoyuan 333 Taiwan; ^7^ Chang Gung University 259, Wenhua 1st Rd. Taoyuan 333 Taiwan; ^8^ Department of Surgery and Liver Research Center Chang Gung Memorial Hospital 5, Fuxing St. Taoyuan 333 Taiwan; ^9^ Institute of Cellular and Organismic Biology Academia Sinica 128, Section 2, Academia Rd. Taipei 115 Taiwan; ^10^ Genomics Research Center Academia Sinica 128 Section 2, Academia Rd. Taipei 115 Taiwan; ^11^ Department of Pediatrics University of California San Diego 9500 Gilman Dr La Jolla CA 92093 USA

**Keywords:** Adenosine receptor 2A, Globo H ceramide, TRAX, Treg

## Abstract

Adenosine signaling is a crucial immunosuppressive pathway within the tumor microenvironment, making it a promising target for cancer therapy. In this study, it is demonstrated that Globo H ceramide (GHCer), the most prevalent tumor‐associated glycosphingolipid, influences the tumor microenvironment by activating adenosine signaling, which results in dual immunosuppressive effects on T cells. It is demonstrated that GHCer interacts with the adenosine receptor 2A (A2AR), triggering cyclic AMP (cAMP) and protein kinase A (PKA) signaling. This interaction leads to a reduction in the proliferation of CD4^+^ T cells while simultaneously promoting the differentiation of regulatory T cells (Tregs). Furthermore, GHCer enhances the suppressive capacity of Treg cells by upregulating inhibitory molecules such as Lymphocyte‐activation gene 3 (LAG3), cytotoxic T‐lymphocyte‐associated protein 4 (CTLA‐4), Programmed cell death 1 ligand 1 (PD‐L1), and it stimulates the secretion of the immunosuppressive cytokine Interleukin 35 (IL‐35). Additionally, GHCer‐induced Tregs express CD39 and CD73, which further enhances adenosine production and creates a positive feedback loop in the adenosinergic pathway and A2AR signaling. Mechanistically, it is found that GHCer forms a complex with TRAX (translin‐associated factor‐X) and the C‐terminus of A2AR, which facilitates the activation of A2AR and promotes an immunosuppressive tumor microenvironment.

## Graphical abstract

This study identifies Globo H ceramide (GHCer) as a novel immune checkpoint molecule that suppresses the activation of conventional T lymphocytes while promoting the differentiation and function of regulatory T cells. The immunosuppressive effects of GHCer are mediated through the activation of the A2AR/cAMP/PKA pathway, which involves its interactions with both TRAX and A2AR.

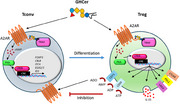

## 1 Introduction

In our previous study, we demonstrated that GHCer shed from cancer cells was taken up by tumor‐infiltrating lymphocytes, leading to the down‐regulation of Notch1 in these lymphocytes. This downregulation helps cancer cells evade immune surveillance.^[^ ^1^ ^]^ Notch signaling is essential for optimal T cell activation and function^[^ ^2^ ^]^; its downregulation enhances the survival and suppressive activity of regulatory T cells (Tregs).^[^ ^3^, ^4^ ^]^ Therefore, GHCer may play a significant role in Treg differentiation and function.

Recent studies have shown that T cell receptor‐induced Notch1 activation is inhibited by A2AR stimulation.^[^ ^5^ ^]^ Furthermore, activating A2AR on naive CD4^+^ T cells promotes the development of Treg cells.^[^ ^6^ ^]^ A2AR, a member of the adenosine receptor family, is the predominant adenosine receptor expressed in T cells.^[^ ^7^ ^]^ Its ligand, extracellular adenosine, is generated from ATP degradation by the CD39/CD73 ectonucleotidases, which are highly expressed in Treg cells.^[^ ^8^ ^]^ Several studies have reported that the accumulation of adenosine in the tumor microenvironment suppresses antitumor immunity.^[^ ^9^, ^10^, ^11^, ^12^, ^13^ ^]^

Activation of A2AR has been shown to increase the number of Treg cells and enhance their immunoregulatory functions.^[^ ^6^ ^]^ Treg cells employ primary mechanisms of suppression: (1) Inhibitory surface molecules, including PD‐1, CTLA‐4, LAG3^[^ ^2^, ^14^ ^]^; (2) Soluble mediators, including interleukin‐10 (IL‐10), transforming growth factor β (TGF‐β),^[^ ^15^ ^]^ IL‐35,^[^ ^16^ ^]^ and adenosine.^[^ ^6^ ^]^

Activation of A2AR triggers the production of cAMP, which subsequently activates downstream PKA and cAMP response element‐binding protein (CREB).^[^ ^17^ ^]^ Additionally, the interaction between the C‐terminus of A2AR and TRAX is essential for A2AR signaling.^[^ ^18^ ^]^ Our previous findings indicated that GHCer‐induced endothelial cell activation is mediated through its interaction with TRAX.^[^ ^19^ ^]^ This suggests that GHCer‐TRAX‐A2AR may play a crucial role in modulating A2AR signaling.

In the study, we demonstrate that the immunosuppressive effects of GHCer are mediated by the A2AR/cAMP/PKA pathway. Evidence from A2AR‐knockout mice further supports this conclusion. We further explored the molecular interactions among GHCer, TRAX, and A2AR in vitro, revealing that GHCer enhances the association between A2AR and TRAX, facilitating immunosuppression at the cellular level.

## 2 Results

### 2.1 GHCer Promotes the Differentiation of Treg and Enhances Its Immunosuppressive Activity

**Figure 1:**
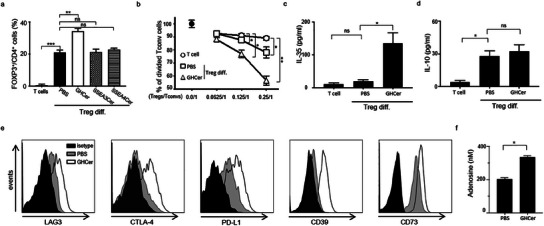
GHCer promotes the differentiation of Treg cells and enhances its immunosuppressive activity. a) GHCer increases Treg differentiation from T cells of healthy donors. Human CD4^+^ T cells were cultured in a Treg differentiation medium containing anti‐CD3 and anti‐CD28 monoclonal antibodies, IL‐2, and TGF‐β in the presence of 30 µM GHCer, SSEA3Cer, SSEA4Cer, or PBS for 6 days. CD4^+^ T cells cultured in PBS for 6 days without activation by anti‐CD3 and anti‐CD28 antibodies were used as a control for Treg differentiation. b) GHCer‐induced Treg cells exhibit greater suppression of the proliferation of Tconv cells. Suppressive activities of human CD4^+^CD25^+^ Treg induced in the presence of GHCer or PBS after washing twice were assessed by incubating with CFSE‐labelled, anti‐CD3/CD28 activated CD4^+^CD25^−^ T cells (Tconv) at the indicated ratios. At 72 h, the proliferation of Tconv was analyzed by FACS. The Tconv cells without Treg cells were used as a control for normalization, represented by 100% (a black circle). The percentage of proliferating cells was normalized against the Tconv cells only. c) IL‐35 in the supernatants from GHCer‐induced Treg cells was measured using an IL‐35 Sandwich ELISA. d) IL‐10 production in supernatants from GHCer‐induced Treg cells was measured using an IL‐10 Sandwich ELISA. e) Flow cytometry analysis of LAG3, CTLA‐4, PD‐L1, CD39, and CD73 expression on human Treg cells induced in PBS or GHCer. f) Adenosine concentration in the supernatants of differentiated Treg on day 6. Supernatants from Treg differentiation were filtered through a 0.22 µm filter. Adenosine in the supernatant was measured by colorimetric assay. Data represent three experiments, and values are expressed as means ± SD. Statistical significance was calculated using ANOVA with Tukey correction for multiple comparisons. ∗p < 0.05; ∗∗p < 0.01; ***p < 0.001.

We previously demonstrated that GHCer shed from tumor cells suppresses the activation of T and B lymphocytes.^[^ ^1^ ^]^ To evaluate the effects of GHCer on Treg differentiation, human CD4^+^ T cells were incubated with TGF‐β (5 ng ml^−1^), IL‐2 (100 U ml^−1^), anti‐CD3 (1 µg ml^−1^), and anti‐CD28 (5 µg ml^−1^) antibodies for 6 days, along with 30 µM of either globosides, GHCer, SSEA3Cer (SSEA3 cermide), SSEA4Cer (SSEA4 cermide), or PBS as a control. As shown in **Figure** **1**a, GHCer significantly increased the proportion of Treg cells (CD4^+^CD25^+^FOXP3^+^) from 21.2% in the PBS control group to 34.4% (p < 0.01). In contrast, no significant changes were observed with SSEA3Cer (20.9%) or SSEA4Cer (22.9%). These results indicate that the immunomodulatory activity of GHCer is dependent on its glycan structure. Next, we assessed the suppressive function of GHCer‐induced Treg cells on the proliferation of conventional T cells (Tconv, CD4^+^CD25^−^). Treg cells generated in PBS inhibited the proliferation of Tconv cells in a dose‐dependent manner, to 88 ± 1% (p < 0.05) and 78 ± 4% (p < 0.05) of the control (Tconv alone) at Treg:Tconv ratios of 0.125:1 and 0.25:1, respectively (Figure 1b). In comparison, GHCer‐induced Treg cells caused a more significant reduction in Tconv proliferation to 77 ± 3% (p < 0.05) and 57 ± 3% (p < 0.01), respectively. In short, GHCer increased both the number and suppressive capacity of Treg cells.

Next, we explored the soluble factors and surface molecules that might enhance the functions of GHCer‐induced Treg cells.^[^ ^20^ ^]^ Notably, GHCer‐induced Treg cells exhibited higher gene expression levels of interleukin‐27 and *IL‐35* (*EBI3* and *IL‐35P35)* but lower interleukin‐10 (*IL‐10*) levels compared to control Treg cells (Supplementary Figure S1, Supporting Information). Correspondingly, the concentration of IL‐35 cytokine in the culture supernatant of GHCer‐induced Tregs was significantly higher than in control Tregs (19.0±4.7 vs 136.3±31.2 pg ml^−1^, p < 0.05, Figure 1c), while no significant difference was observed for IL‐10 (28.3 ± 4.5 vs 32.6±5.9 pg ml^−1^, Figure 1d). Flow cytometry analyses further revealed that GHCer‐induced Treg cells exhibited higher surface expression of LAG3, CTLA4, and PDL1 than control Treg cells (Figure 1e). This finding aligns with previous reports indicating that Tregs expressing inhibitory receptors possess a greater immunosuppressive capacity.^[^ ^21^, ^22^, ^23^ ^]^ Additionally, GHCer‐induced Tregs showed greater surface expression of ectonucleotidases CD39 and CD73 than control Tregs (Figure 1e), consistent with reports that CD39^+^CD73^+^ Treg exhibits enhanced suppressor function.^[^ ^24^, ^25^ ^]^ Given that CD39 and CD73 could convert ATP to adenosine, a critical immunosuppressive mediator generated by Tregs, we found a significantly higher concentration of adenosine in supernatants from GHCer‐induced Treg cells (334.7 ± 9.4 nM) compared to control Treg cells (203 ± 7.8 nM, p <0.01) (Figure 1f). To sum up, the presence of GHCer during the induction of Treg differentiation significantly increased both the number and the suppressive activity of FOXP3^+^ Treg cells, along with the expression of several inhibitory mediators, including IL‐35, LAG3, CTLA‐4, PD‐L1, CD39, CD73, and adenosine. These findings suggest that the immunosuppressive activities of GHCer may be attributed to the activation of the adenosinergic pathway, highlighting the critical roles of Treg cells, CD39, and CD73 in adenosine generation.

### 2.2 Immunosuppression Induced by GHCer is Mediated by the Activation of A2AR/cAMP/PKA Pathway

**Figure 2:**
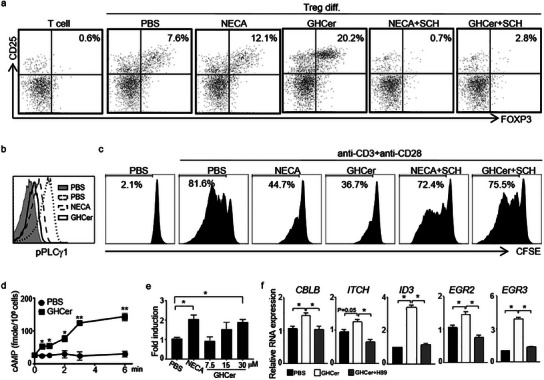
GHCer‐induced immunosuppression is mediated by activating the A2AR/cAMP/PKA pathway. a) Effects of GHCer or A2AR agonist NECA on Treg differentiation from T cells of healthy donors. CD4^+^ T cells from human PBMC were cultured in a Treg differentiation medium in the presence of PBS, 1 µM NECA (A2AR agonist), or 30 µM GHCer and in the presence or absence of 20 µM A2AR antagonist SCH58261 (SCH) for 6 days and analyzed by FACS. b) Effects of GHCer or A2AR agonist NECA on TCR activation in T cells. Primary CD4^+^ T cells were incubated in the presence or absence of 30 µM GHCer for 24 h and then activated with anti‐CD3 and anti‐CD28 antibodies for 10 min. Cells were fixed, permeabilized, and stained with ant‐phospho‐PLCγ1 (Tyr783)‐PE. c) Effects of GHCer or A2AR agonist NECA on the proliferation of T cells from healthy donors. Human CD4^+^ T cells were isolated and labeled with CFSE, then stimulated with anti‐CD3 and anti‐CD28 antibodies in the presence of 1 µM NECA or 30 µM GHCer with or without SCH. T cells are analyzed by FACS after 72 h. d) Effect of GHCer on intracellular cAMP accumulation of T cells. CD4^+^ T cells (10^6^ cells ml^−1^) were incubated with the 30 µM GHCer. At the indicated time, T cells were lysed using the LANCE cAMP Assay to measure cAMP levels. e) cAMP response element binding protein (CREB) reporter activities of GHCer‐treated Jurkat cells. A dual‐luciferase reporter assay measured the effects of GHCer on the activation of CREB. Jurkat cells were transfected with the pGL4.29 (luc2P/CRE/Hygro) expression vector of the CRE firefly luciferase reporter and Renilla luciferase and cultured in serum‐free medium for 4 h before incubation for 6 h with the indicated concentrations of GHCer or NECA. The firefly luciferase activities were normalized to those of Renilla luciferase. The luciferase activity of whole‐cell lysates was determined by luminometry and normalized to the control in PBS control. (f) RT‐qPCR analysis of anergy genes (*CBLB, ITCH, ID3, EGR2, EGR3*) in GHCer‐treated cells. Primary CD4^+^ T cells were incubated with GHCer in the presence or absence of 100 µM H89 for 24 h and then activated by anti‐CD3 and CD28 antibodies. Total RNA was extracted 2 h after activation to quantify the expression of *EGR2* and *EGR3* and at 12 h for *CBLB*, *ITCH*, and *ID3* using real‐time quantitative PCR. The mRNA levels were normalized to the level of *GADPH* (Glyceraldehyde 3‐phosphate dehydrogenase) and shown as a fold change compared to the resting cell. The data are presented as the mean ± SD of three replicates; a comparison was made between GHCer with and without H89 (100 µM). Statistical significance was calculated using ANOVA with Tukey correction for multiple comparisons. *p < 0.05, **p < 0.01.

Since adenosine signaling through the A2a receptor expressed on immune cells potently dampens immune responses,^[^ ^26^ ^]^ we next investigated the possible involvement of the A2AR pathway in GHCer‐induced Treg differentiation. We first compared the effects of an agonist of A2AR, 1 µM 5′‐N‐Ethylcarboxamidoadenosine (NECA), and 30 µM GHCer on Treg differentiation. As shown in **Figure** **2**a, NECA and GHCer enhanced the relative abundance of Treg cells from 7.6% in PBS control to 12.1% and 20.2%, respectively. The effects of NECA and GHCer were abolished by the A2AR antagonist, SCH58261 (SCH), at 20 µM. These findings suggest that GHCer may promote Treg differentiation through A2AR signaling. In addition to promoting Treg differentiation, GHCer blocked the activation of conventional T cells as reported previously^[^ ^1^ ^]^ and was confirmed by reduced TCR‐activation‐induced phosphorylation phospholipase Cγ1 (PLCγ1) at Tyr783 (Figure 2b). Thus, we examined the possibility that GHCer‐induced suppression of conventional T cell activation may also involve A2AR signaling.^[^ ^1^ ^]^ Incubation of human CD4^+^ T cells with 30 µM GHCer or 1 µM NECA for 24 h before activation by anti‐CD3 (1 µg ml^−1^) and anti‐CD28 (5 µg ml^−1^) antibodies resulted in a marked reduction of CD4^+^ T cell proliferation from 81.6% (PBS control) to 36.7%, and 44.7%, respectively (Figure 2c). Additionally, the suppressive effects of GHCer and NECA were abolished by the A2AR antagonist (SCH), as shown in Figure 2c. These results indicate that the suppression of conventional T‐cell activation induced by GHCer is mediated through the A2AR signaling pathway.

Next, we investigated the impact of GHCer on the downstream signaling of A2AR, which involves increased cAMP production, leading to the consequent activation of PKA and CREB. Incubation of CD4^+^ T cells with 30 µM GHCer resulted in a steady, time‐dependent increase in cAMP concentration (Figure 2d). GHCer‐induced cAMP signaling activates CREB, as demonstrated by a dual‐luciferase reporter assay using Jurkat cells transfected with a CRE‐Luc reporter gene cassette. As expected, treatment of Jurkat cells with 1 µM NECA increased luciferase activity to 2.3‐fold over the basal level (p = 0.02). GHCer induced a dose‐dependent increase in CRE‐Luc luciferase activity in Jurkat cells, reaching a 2.1‐fold increase over baseline (p = 0.02) at 30 µM, comparable to that induced by NECA (Figure 2e). These results further confirm the roles of GHCer in activating the A2AR pathway.

In our previous work, we demonstrated that GHCer downregulated *NOTCH1* expression at the transcriptional level by upregulating co‐repressor *ID3* and at the translational level by upregulating Early growth response protein 2/3 (*EGR2/3*). The latter subsequently raised the expression of E3 ligases, including *ITCH* and *CBLB*.^[^ ^1^ ^]^ However, the upstream signaling pathway involved remained unclear. As the expression of *EGR2/3*, the transcriptional factors mediating T cell anergy,^[^ ^27^, ^28^ ^]^ is regulated by the c‐AMP/PKA/CREB pathway,^[^ ^27^, ^29^, ^30^, ^31^ ^]^ we investigated whether the PKA inhibitor, H‐89, could suppress GHCer‐induced upregulation of *CBLB*, *ITCH*, *ID3*, and *EGR2/3*. Primary CD4^+^ T cells were incubated with 30 µM GHCer for 24 h before being activated with anti‐CD3/CD28 monoclonal antibodies (mAbs). Total RNA was extracted for gene expression analysis by real‐time quantitative PCR. As shown in Figure 2f, the expression of *CBLB*, *ITCH*, *ID3*, *EGR2*, and *EGR3* was upregulated 1.5‐, 1.3‐, 3.4‐, 1.5‐, and 3.9‐fold, respectively, compared to the PBS control. In contrast, adding 100 µM H89 reduced the expression of these GHCer‐upregulated genes to 1.0‐, 0.7‐, 1.1‐, 0.8‐, and 1.3‐fold, respectively, of the PBS control. These results indicate that the PKA inhibitor reduced the upregulation of these anergy‐related genes to 67%, 54%, 32%, 53%, and 33% of the levels observed in GHCer‐treated cells, respectively. These findings confirm that the immunosuppressive activity of GHCer is associated with the upregulation of anergy‐related genes through PKA activation (Figure 2f). Our results suggest that GHCer promotes immunosuppression by enhancing Tregs and inhibiting Tconv cells, mediated by A2AR signaling, which leads to increased intracellular cAMP and subsequent activation of PKA and CREB.

### 2.3 GHCer‐Induced Immunosuppression is A2AR‐Dependent

**Figure 3:**
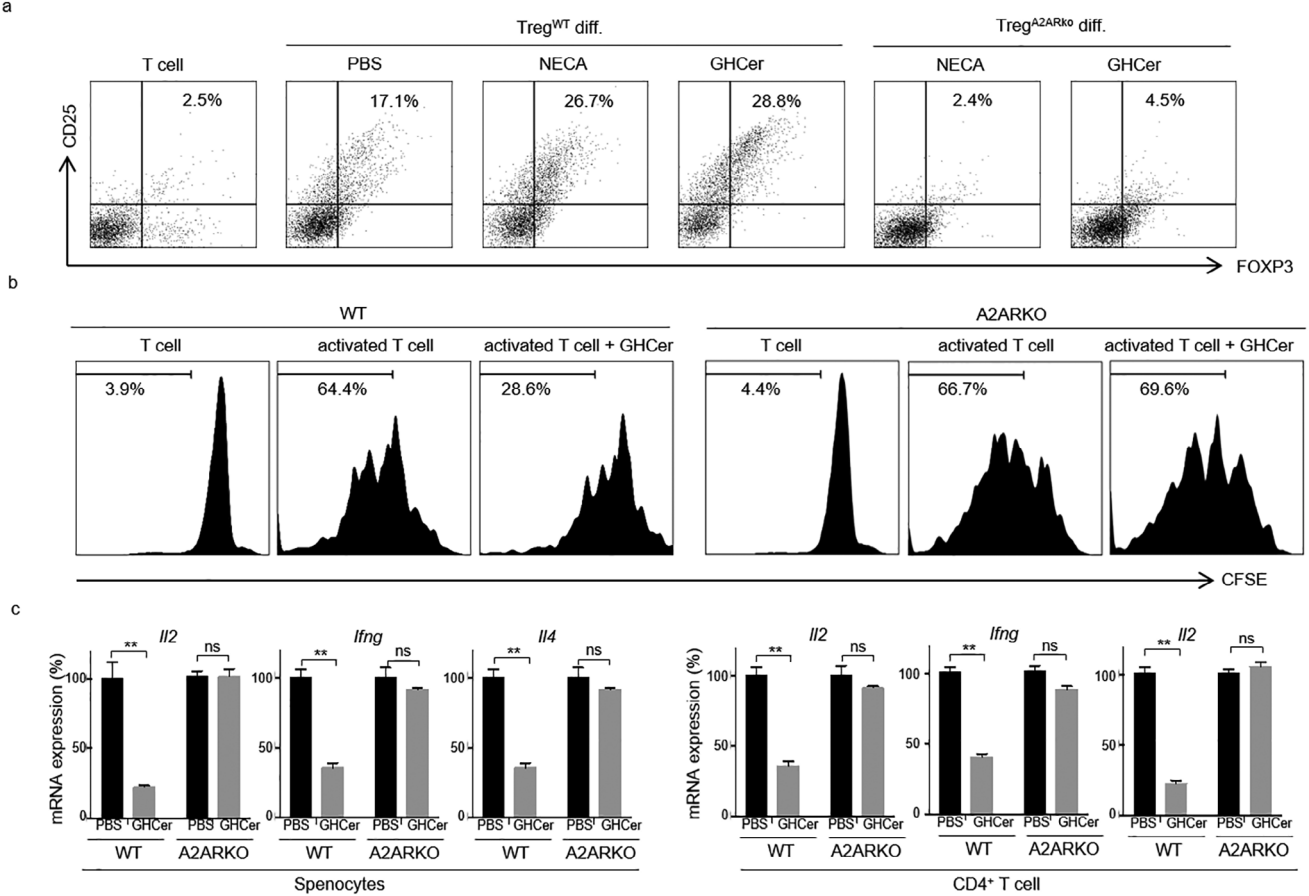
GHCer‐mediated immunosuppression is A2AR dependent. a) Effects of GHCer or A2AR agonist NECA on Treg differentiation of T cells from A2ARKO mice. CD4^+^ T cells isolated from wildtype (WT) or A2ARKO mice were cultured in a Treg differentiation medium in the presence of PBS, 1 µM NECA, or 30 µM GHCer for 6 days and analyzed by FACS. b) Effects of GHCer or NECA on the proliferation of T cells from A2ARKO mice. CD4^+^ T cells isolated from WT or A2ARKO mice were labeled with CFSE and stimulated with anti‐CD3 and anti‐CD28 antibodies in the presence of GHCer. At 72 h, T cell proliferation was analyzed by FACS. c) The qPCR analysis of activated genes (*Il2, Ifng, Il4*) in GHCer‐treated cells. Mouse splenocytes or CD4^+^ T cells were incubated with 30 µM GHCer or PBS for 24 h and then activated anti‐CD3 and CD28 antibodies for 16 h. RNA was collected to determine *Il2*, *Ifng*, and *Il4* expression. The mRNA levels were normalized to the level of *β‐actin* and shown as fold change relative to control cells treated with PBS. Data represent three experiments, and values are expressed as means ± SD. Statistical significance was calculated using ANOVA with Tukey correction for multiple comparisons. ∗∗p < 0.01.

To further assess the A2AR dependency of the immunosuppressive activity of GHCer, we utilized a murine system, comparing the effects of GHCer on T cells harvested from wild‐type (WT) and A2AR knockout (A2ARKO) mice. Consistent with the findings in human Treg cells, adding 30 µM GHCer or 1 µM NECA significantly increased the relative abundance of Foxp3‐expressing Treg in CD4^+^ T cells from WT mice (**Figure** **3**a). The GHCer‐induced mouse Treg cells also express higher levels of LAG3, CTLA‐4, PD‐1, and IL‐35 than the control Treg cells (Supplementary Figure S2, Supporting Information). In contrast, GHCer failed to promote Treg differentiation of the A2ARKO CD4^+^ T cells, mirroring the expected ineffectiveness of NECA (Figure 3a). Thus, GHCer‐induced Treg differentiation is contingent upon the presence of A2AR.

As to Tconv cells, GHCer inhibited the proliferation of murine T cells as effectively as human T cells (Figure 3b). However, it failed to suppress the proliferation of A2ARKO T cells, indicating that A2AR is also essential for the inhibitory effect of GHCer on T cell activation and proliferation. To further investigate the impact of GHCer on the cytokine production of activated T cells, we pretreated murine splenocytes or purified CD4^+^ T cells from WT or A2ARKO mice with GHCer for 24 h before activation with anti‐CD3 and anti‐CD28 antibodies. At 16 h post‐activation, we performed qPCR to assess the mRNA expression levels of *Il2*, *Ifng*, and *Il4*. As shown in Figure 3c, GHCer reduced *Il2* expression in activated splenocytes or CD4^+^ T cells from WT mice to 19 ± 1% and 13 ± 3%, respectively, of control levels, with no effect on A2ARKO cells. GHCer also inhibited *Ifng* expression to 40 ± 3% and 39 ± 2%, and *Il4* expression to 28 ± 2% and 22 ± 1% in WT splenocytes or CD4^+^ T cells, respectively, but did not affect A2ARKO cells. Collectively, these data from A2ARKO mice further support the notion that the immunosuppressive activities of GHCer characterized by the promotion of Treg differentiation and inhibition of Tconv activation, proliferation, and cytokine production, are indeed A2AR dependent.

### 2.4 GHCer Activates A2AR Signaling by Increasing the Interactions Between A2AR and TRAX

**Figure 4:**
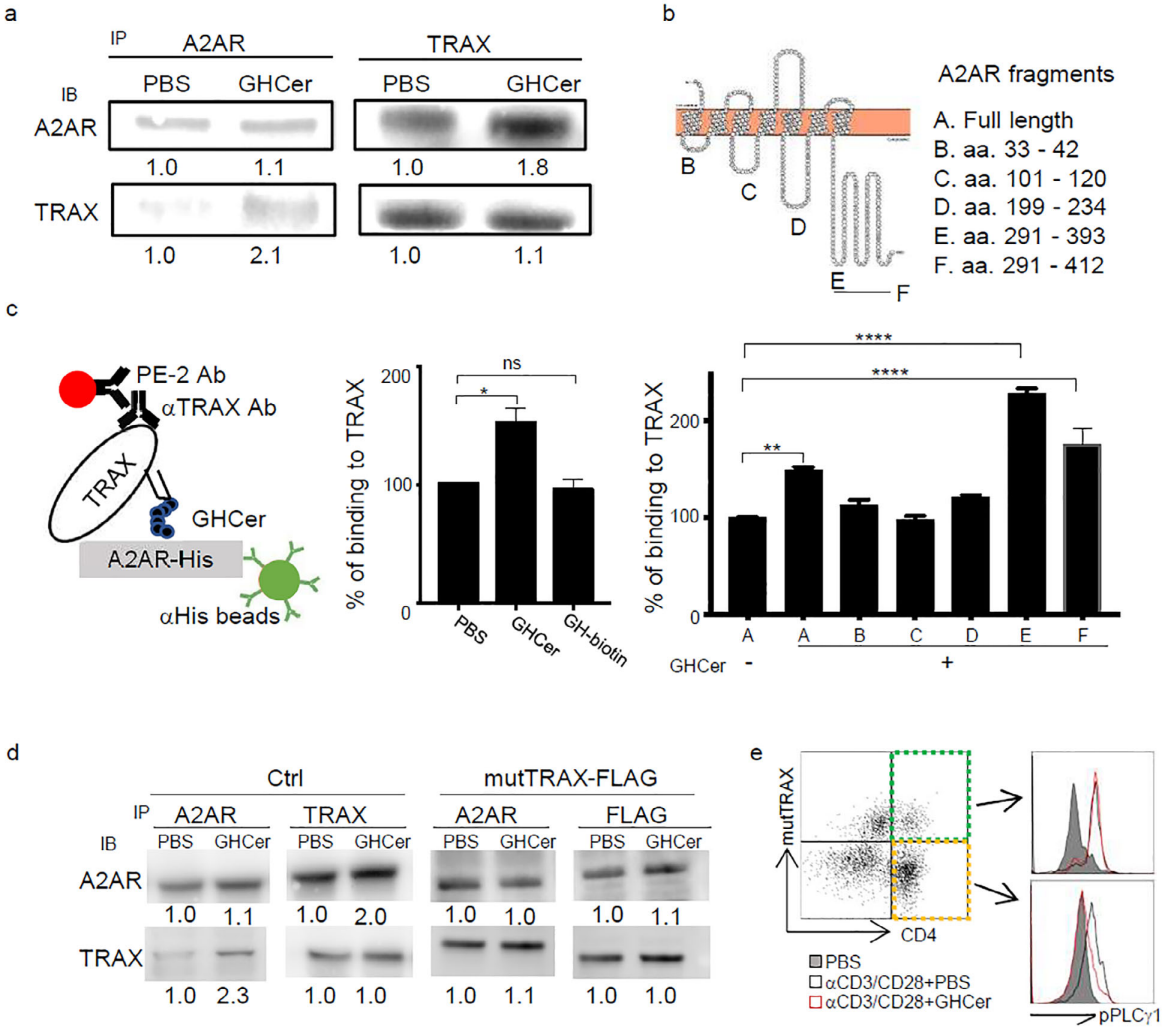
GHCer enhances A2AR downstream signaling by facilitating the interaction between TRAX and A2AR. a) Co‐immunoprecipitation of A2AR‐GHCer‐TRAX complex in CD4^+^ T cells. Human primary T cell lysates incubated with GHCer were immunoprecipitated with anti‐A2AR or anti‐TRAX antibodies. The immunoprecipitates were subjected to western blot for probing with anti‐TRAX antibody or anti‐A2AR antibody, respectively. b) The diagram of A2AR depicts its secondary structure, denoting various fragments used in the following experiments. c) Molecular interactions of A2AR‐GHCer‐TRAX complex. Schema (left) of Luminex bead‐based assay to measure binding of TRAX to beads conjugated with A2AR‐full length (middle) or fragments (right) in the presence of PBS or 30 µM Globo H‐biotin (GH‐biotin) or GHCer. d) Co‐immunoprecipitation of A2AR‐GHCer‐mutTRAX complex in Jurkat cells. Jurkat‐vector cell lysates (left panel) and Jurkat‐mutTRAX‐FLAG (right panel) incubated with GHCer were immunoprecipitated with anti‐A2AR or anti‐TRAX antibodies. The immunoprecipitates were subjected to western blot for probing with anti‐TRAX antibody or anti‐A2AR antibody, respectively. e) Effect of GHCer on TCR activation in mutTRAX‐T cells. Primary CD4 T cells transduced with mutTRAX‐FLAG lentivirus were incubated in the presence or absence of 30 µM GHCer for 24 h and activated by anti‐CD3 and anti‐CD28 antibodies for 10 min. Cells were fixed, permeabilized, and stained with ant‐phospho‐PLCγ1 (Tyr783)‐PE. The green gate represented the mutTRAX‐T cells, and the yellow gate represented the wild‐type TRAX‐T cells. Data represent more than three experiments; values are expressed as mean ± SD. Statistical significance was calculated using ANOVA with Tukey correction for multiple comparisons. ∗p < 0.05; **p < 0.01; ***p < 0.001; ****p < 0.0001.

To elucidate the molecular mechanism of GHCer‐induced A2AR signaling, we built on our previous discovery that the angiogenic activity of GHCer was mediated through its interaction with TRAX in endothelial cells.^[^ ^19^, ^32^ ^]^ Additionally, it has been reported that TRAX bind to A2AR, which is necessary for A2AR activation in neuronal cells.^[^ ^33^, ^34^ ^]^ Thus, we investigated whether the immunosuppressive effects of GHCer may involve its interaction with TRAX and A2AR. We incubated human CD4^+^ T cells with 30 µM GHCer at 37 °C for 1 h, followed by immunoprecipitation using mAbVK9. Indeed, both A2AR and TRAX were detected in the immunoprecipitates. Notably, in the presence of the GHCer, immunoprecipitation with anti‐A2AR successfully pulled down 2.1‐fold more TRAX than the PBS control (**Figure** **4**a), while anti‐TRAX also pulled down 1.8‐fold more A2AR than the control (Figure 4a). These co‐IP studies suggest that GHCer may act as a facilitator of the TRAX and A2AR interaction.

Using computer modeling and molecular dynamics simulation, we previously demonstrated that the ceramide tail of GHCer binds to the hydrophobic groove on TRAX, and the glycan moiety resides on the protein surface.^[^ ^19^, ^32^ ^]^ This configuration likely allows the glycan moiety to interact with nearby proteins. Given the known interaction between TRAX and A2AR, we hypothesized that the glycan moiety of GHCer enhances the interaction between TRAX and A2AR. To test this, we performed a Luminex‐based protein‐protein interaction to delineate the binding site of the glycan moiety. We cloned various fragments of the intracellular loops of A2AR: A. full‐length, B. aa 33–42, C. aa 101–120, D. aa 199–234, E. aa 291–393, and F. aa 291–412 (Figure 4b). The genes encoding TRAX and A2AR fragments were expressed in *Escherichia coli* to produce His‐tagged recombinant proteins. Luminex beads, conjugated with anti‐His antibody, captured A2AR‐His proteins for interaction assays. The interactions of various fragments of A2AR with TRAX, 30 µM GHCer, or 30 µM Globo H‐biotin (GH‐biotin, the glycan moiety of GHCer conjugated with biotin) were detected by specific antibodies or streptavidin PE (SA‐PE), respectively (detailed diagram in Figure 4c). Coating the beads with full‐length recombinant A2AR protein (A2AR‐His, fragment A) revealed that GHCer but not GH‐biotin enhanced binding of TRAX to A2AR by 1.6‐fold of PBS control (Figure 4c), underscoring the importance of the ceramide moiety of GHCer for complex formation. Next, the binding signals of TRAX to beads coated with these A2AR were normalized to full‐length A2AR/PBS/TRAX binding signals. The presence of GHCer increased the binding of TRAX to fragments A, E, and F by 1.5, 2.3, and 1.8 folds, respectively, of fragment A without GHCer (Figure 4c), confirming that TRAX binds to full‐length A2AR, with GHCer promoting this interaction by binding to the C‐terminal region of A2AR.

In our recent study, mutations Q219A and Q223A in TRAX abrogated its binding to GHCer.^[^ ^32^ ^]^ Although binding of TRAX to A2AR is necessary for A2AR activation in neuronal cells,^[^ ^33^, ^34^ ^]^ its role in GHCer‐mediated A2AR signaling remains unclear. To investigate this, we transfected Jurkat cells with FLAG‐tagged TRAX^Q219A, Q223A^ (Jurkat‐mutTRAX‐FLAG), or vector‐only lentivirus (Jurkat‐vector). Following incubation with 30 µM GHCer or PBS at 37 °C for 1 h, we conducted immunoprecipitations using anti‐A2AR and anti‐TRAX antibodies. In Jurkat‐vector, immunoprecipitation with anti‐A2AR revealed a 2.3‐fold increase in TRAX pull‐down with GHCer over the PBS control (Figure 4d, left panel), while immunoprecipitation with anti‐TRAX also pulled down 2.0‐fold more A2AR than the control (right panel), consistent with the findings in the primary T cell results (Figure 4a). In contrast, the GHCer failed to enhance complex formation in Jurkat‐mutTRAX‐FLAG, which showed no increase in anti‐A2AR pulled down (1.0‐fold of the PBS control) or anti‐FLAG pulled down (1.1‐fold over the control) (Figure 4d, right panel). Similarly, GHCer suppresses pPLCγ1 in FLAG^−^CD4^+^ primary T cells (yellow panel, Figure 4e) but not FLAG^+^ primary T cells (green panel, Figure 4e). These findings indicate that TRAX is required for GHCer‐induced A2AR signaling.

### 2.5 GHCer Binds to the C‐Terminus of A2AR Through Its Glycan Moiety

**Figure 5:**
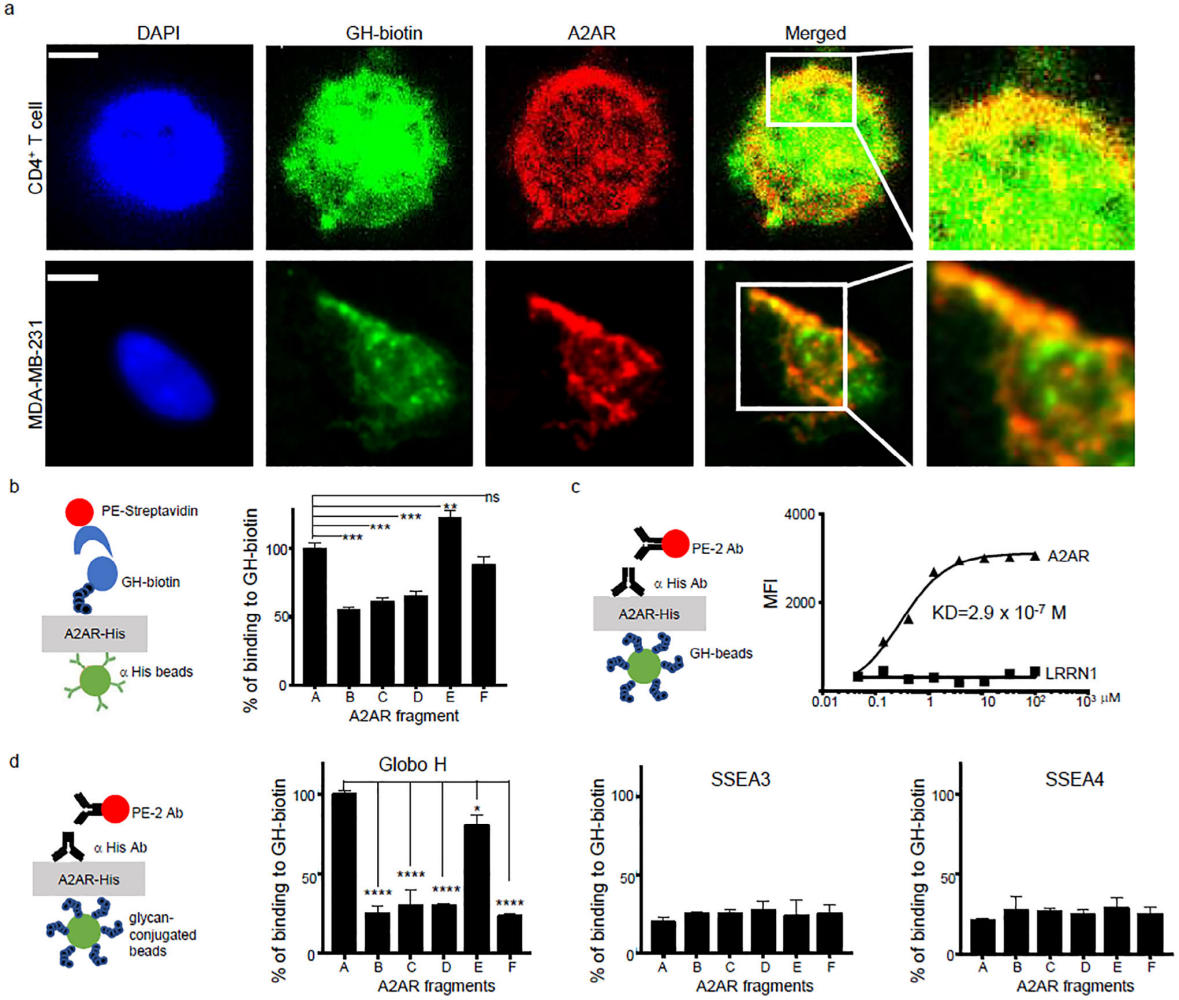
GHCer binds to the C‐terminus of A2AR through its glycan moiety. a) Colocalization (yellow) of GH‐biotin (green) and A2AR (red) in A2AR^+^ cells. Cells were fixed and permeabilized, then incubated with GH‐biotin. Cells were stained with streptavidin‐conjugated Alexa Flour 488 and anti‐A2AR antibody. The nucleus was stained with DAPI. The scale bar is 5 µm). b) Luminex analysis of GH‐biotin binding to A2AR. Schema (left) of Luminex bead‐based assay to measure binding of GH‐biotin to beads conjugated with A2AR fragments (right). c) The binding affinity of Globo H to A2AR or an unrelated protein LRRN1. Schema (left) of Luminex bead‐based assay to measure binding of A2AR and LRRN1 to Globo H conjugated beads (right). d) Binding specificity of Globo H and related glycans to A2AR. Schema (left) of Luminex bead‐based assay to measure binding of A2AR fragments to beads coated with 1 µg Globo H, SSEA3, or SSEA4 by click reaction (right). Data represent more than three experiments; values are expressed as mean ± SD. Statistical significance was calculated using ANOVA with Tukey correction for multiple comparisons. ***p < 0.001; ****p < 0.0001.

To further examine the interaction of glycan moiety of GHCer with A2AR at the cellular level, we examined their colocalization in two types of A2AR‐expressing cells, MDA‐MB‐231 and primary human T CD4^+^ cells. After fixation and permeabilization, the cells were treated with anti‐A2AR mAb and GH‐biotin, followed by Alexa Fluor 555 conjugated secondary antibody and Alexa Fluor 488 conjugated streptavidin. The GH‐biotin (green) was distributed in the entire cell, while A2AR (red) showed a more concentrated presence at the plasma membrane in both cell types (**Figure** **5**a). Subsequent immunofluorescence staining revealed the colocalization of GH‐biotin with A2AR in both cell types, which appeared yellow in the merged images. Z‐stack image analyses confirmed the overlap of signals from GH‐biotin and A2AR (Figure 5a).

To decipher the binding of the glycan moiety of GHCer to A2AR, we assessed the binding of GH‐biotin to beads coated with various A2AR fragments (illustrated in Figure 5b). The results indicated that GH‐biotin was specifically bound to fragment E, exhibiting a 1.3‐fold binding signal of full‐length A2AR, while binding to fragment F was comparable to that of full‐length A2AR (Figure 5b). In another assay, beads coated with Globo H (GH‐beads) were incubated with varying concentrations of full‐length N‐His‐A2AR (fragment A, Supplementary Figure S3, Supporting Information) or an unrelated membrane protein, leucine‐rich repeat neuronal protein‐1 (LRRN1), as a control. A2AR demonstrated specific binding to GH‐beads in a dose‐dependent manner, with a dissociation constant (KD) of 2.9 × 10^−7^ M. In contrast, the control protein LRRN1 showed no binding (Figure 5c). In addition, fragments A and E are specifically bound to Globo H conjugated beads but not to beads conjugated with SSEA3 or SSEA4 (Figure 5d). These molecular and cellular studies suggest that the glycan moiety of GHCer facilitates interactions between TRAX and A2AR, thereby activating A2AR signaling.

### 2.6 Tumor‐Infiltrating Treg Cells Correlate with Globo H Expression in Breast Cancer and Hepatocellular Carcinoma

#### 2.6.1 Given our Findings that GHCer Increased the Differentiation of Treg Cells

**Figure 6:**
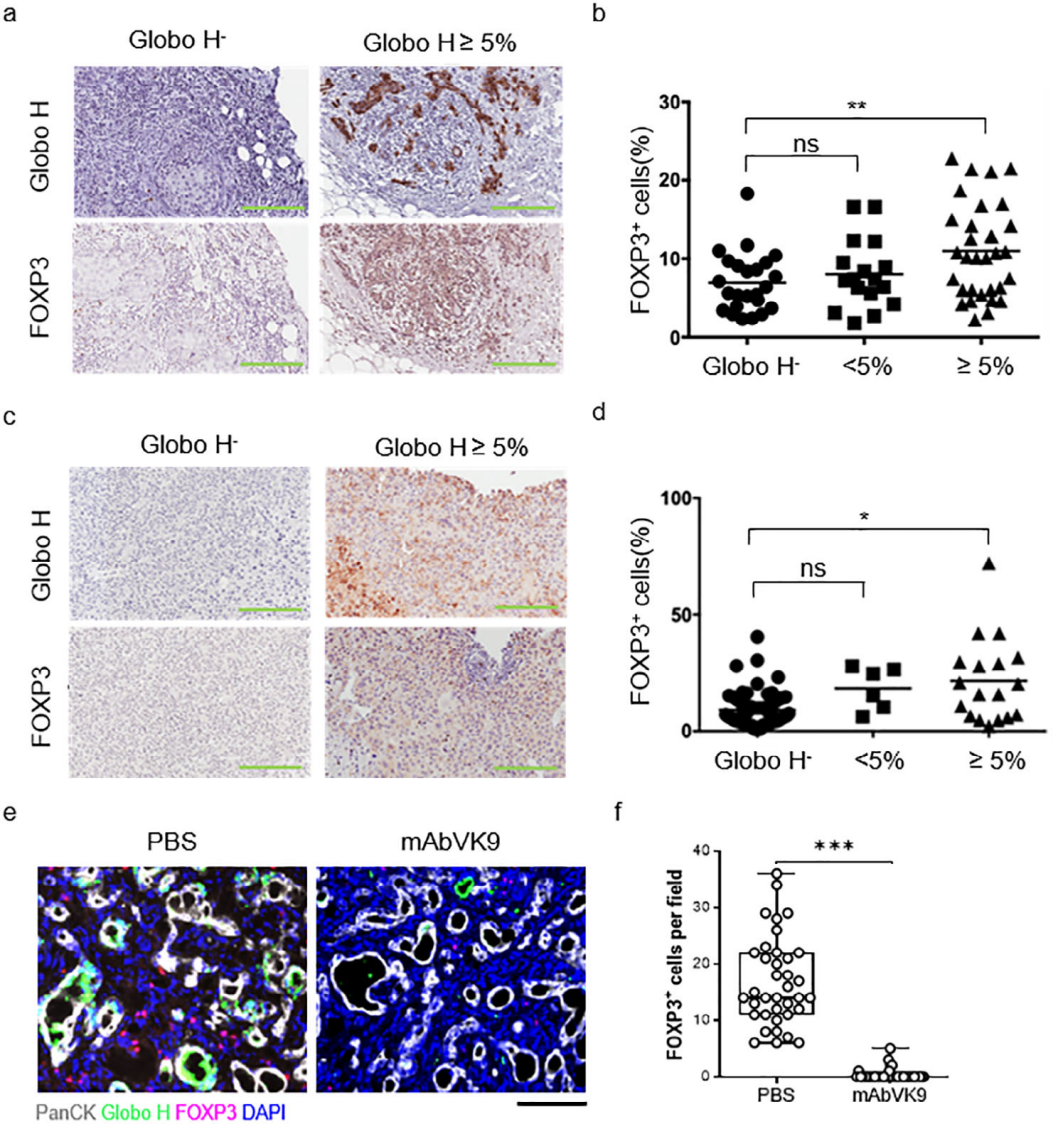
Treg cells correlate with Globo H expression in the tumor microenvironment. a) & c) Immunohistochemical staining for Globo H or FOXP3 in breast and hepatocellular carcinoma. Representative immunostaining for Globo H‐negative (Globo H^−^) and Globo H‐positive ≥5% (Globo H≥5%) tumors was shown. The scale bar is 200 µm. b) & d) Scatterplots showing the frequency of Treg cells within tumor‐infiltrating immune cells in 73 patients with breast cancer and 85 patients with hepatocellular carcinoma, respectively, grouped by their Globo H expression status. Statistical significance was calculated using ANOVA with Tukey correction for multiple comparisons. ∗p < 0.05; ∗∗p < 0.01. e) Representative photomicrographs of a multiplex panel: PanCK (cancer cell marker, grey), Globo H (green), FOXP3 (magenta), and DAPI (blue) from rat intrahepatic cholangiocarcinoma treated with mAbVK9 or PBS control. f) The count of FOXP3^+^ cells per high‐power field in the mAbVK9‐treated and control groups. The scale bar is 100 µm. Statistical analysis was conducted using a t‐test. *** denotes a significant difference from the Ctrl group with a P value < 0.001.

We examined the association between tumor expression of Globo H and the presence of Treg cells as detected by forkhead box protein P3 (FOXP3) expression in clinical specimens from 73 patients with breast cancer (BC) and 85 patients with hepatocellular carcinomas (HCC) (**Figure** **6**a,c). We found that the frequency of tumor‐infiltrating Treg cells was significantly higher in specimens with ≥5% or greater Globo H expression compared to those with <5% or no Globo H expression in both BC (Figure 6b) and HCC (Figure 6d). These results are consistent with the promotion of Treg generation by GHCer.

### 2.7 Treatment with an Anti‐Globo H Antibody Reduces Tumor‐Infiltrating Treg Cells in Rats Bearing Intrahepatic Cholangiocarcinoma

We previously demonstrated that intrahepatic cholangiocarcinoma (ICC) induced by thioacetamide (TAA) in rats exhibits expression of Globo H. Moreover, anti‐Globo H monoclonal antibody (mAbVK9) treatment significantly inhibited tumor growth.^[^ ^35^ ^]^ To further investigate the relationship between Treg cells and Globo H expression in the tumor microenvironment, we generated TAA‐induced ICC in rats and monitored tumor growth using an 18F‐FDG PET/CT scan. As shown in Supplementary Figure S4 (Supporting Information), after 2 months of treatment with mAbVK9, there was a significant reduction in maximum standardized uptake value (SUVmax) compared to the PBS group (fold change: 0.69 vs 1.36, p < 0.01), consistent with the tumor‐suppressive effects of mAbVK9.

Additionally, tumors in the mAbVK9‐treated group exhibited lower Globo H expression (Figure 6e), as expected. Notably, the mAbVK9‐treated group displayed a median of 0 FOXP3^+^ cells per high‐power field (HPF) with a range of 0–3 per HPF), significantly fewer than the PBS group, which had a median of 14 FOXP3^+^ cells per HPF, with a range of 6–36 cells (p < 0.001) (Figure 6f). These findings support the notion that Globo H expression in the tumor microenvironment may contribute to an increased population of Treg cells.

**Figure 7:**
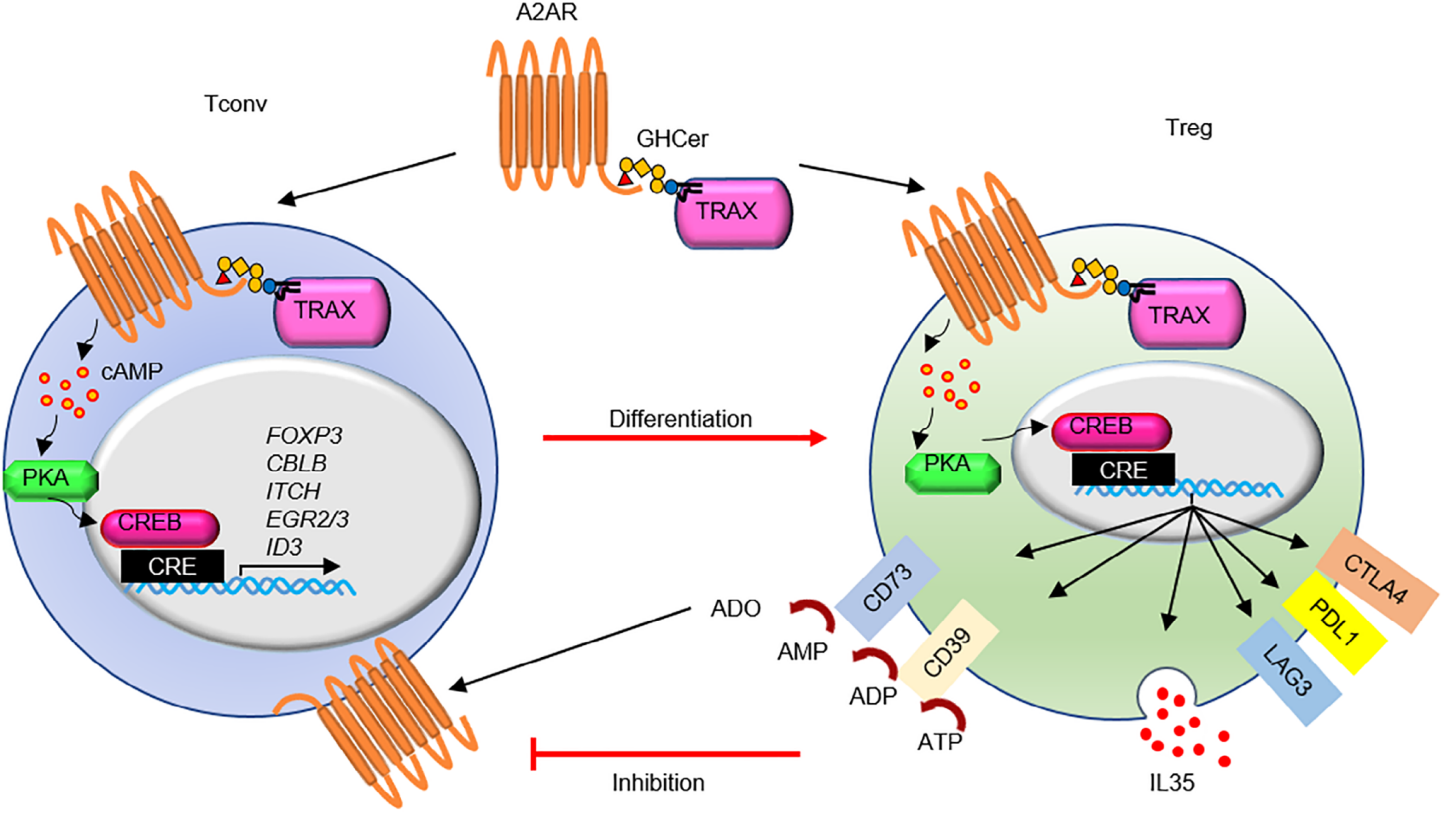
GHCer‐induces A2AR signaling in Tconv/Treg cells by facilitating A2AR and TRAX interaction with increased generation of adenosine. GHCer forms complexes with TRAX and A2AR, triggering A2AR activation in Tconv and Treg cells. In Tconv cells, GHCer stimulates the A2AR to dampen T‐cell activation. At the same time, GHCer promotes Treg expansion and enhances immunosuppressive capacity by upregulating inhibitory surface molecules, IL‐35, and CD39/CD73 ectonucleotidases. The adenosine generated by the latter further activates A2AR signaling, forming a positive feedback loop that contributed to GHCer‐induced immunosuppression.

In conclusion, GHCer forms complexes with TRAX and A2AR, triggering A2AR activation in both Tconv and Treg cells. In Tconv, GHCer activates A2AR signaling to promote T cell anergy. At the same time, GHCer enhances Treg differentiation and immunosuppressive capacity by upregulating inhibitory surface molecules, soluble factors, and ectonucleotidases. The adenosine generated by the latter further activates A2AR signaling, creating a positive feedback loop that promotes GHCer‐induced immunosuppression (**Figure** **7**).

## 3 Discussion

This research is the first to reveal the formation of a complex among A2AR, TRAX, and GHCer. In this complex, the ceramide tail of GHCer binds to a hydrophobic groove of TRAX^[^ ^32^ ^]^ while its glycan head interacts with both TRAX and the cytoplasmic domain of A2AR. Such complex formation is essential for the dual immunosuppressive activities of GHCers. The requirement for A2AR is supported by the loss of GHCer‐mediated immunosuppressive activities when A2AR antagonists are added or in lymphocytes obtained from A2ARKO mice. The critical role of TRAX is demonstrated by the absence of GHCer activities in lymphoid cells expressing TRAX mutants that cannot bind to the glycan moiety of GHCer. We have also provided evidence that the A2AR/GHCer/TRAX complex enhances immunosuppression through A2AR signaling via the downstream cAMP/PKA/CREB pathway.

TRAX is a versatile protein involved in a wide range of cellular functions, including proliferation,^[^ ^36^ ^]^ trafficking of brain‐derived neurotrophic factor (BDNF) mRNA,^[^ ^37^ ^]^ production of RNAi,^[^ ^38^, ^39^ ^]^ neuritogenesis,^[^ ^33^ ^]^ DNA repair,^[^ ^40^ ^]^ and angiogenesis.^[^ ^19^ ^]^ Among its many roles, the interaction between TRAX and glycosphingolipids was not previously identified until our study highlighted its involvement in GHCer‐induced angiogenesis. We demonstrated that the binding of GHCer to TRAX released PLCβ1 sequestered by TRAX, leading to calcium mobilization and subsequent angiogenesis.^[^ ^19^ ^]^ At the molecular level, Biacore assays revealed that GHCer is bound to recombinant TRAX with an apparent dissociation constant of 40.9 nM, but does not bind to TRAX mutants that disrupt the predicted binding site.^[^ ^32^ ^]^ This finding confirms that GHCer does not enhance A2AR signaling in mutant TRAX lymphoid cells.

Glycosphingolipids (GSLs) are important surface membrane glycolipids that communicate with other surface membrane components, contributing to processes such as signaling, receptor trafficking, cell‐cell contact, adhesion, and gene expression.^[^ ^41^, ^42^ ^]^ Additionally, GSLs can be transported to intracellular locations via endocytosis or vesicular trafficking.^[^ ^43^ ^]^ Previously, we demonstrated that exogenously added GHCer could be incorporated into the plasma membrane and transferred to the cytoplasm of HUVEC cells within a few minutes.^[^ ^19^ ^]^ This transfer of GHCer to the cytosol was inhibited by an endocytosis blocker. Similarly, a few studies reported the interaction of GSL with intracellular components. For instance, GM1 gangliosides have been found in the nuclear envelope, where they make direct contact with chromatin and influence the activity of promoters, thereby facilitating the epigenetic activation of neuronal cells.^[^ ^44^ ^]^ On the other hand, GD3 gangliosides are associated with microtubules in mitochondria during T‐cell apoptosis.^[^ ^45^ ^]^ While GSLs are known for their important functions at the plasma membrane, recent research has highlighted their significant roles within cells.

Recently, we showed that SSEA3 ceramide, a pentasaccharide precursor of GHCer lacking terminal fucose, was unable to bind TRAX.^[^ ^32^ ^]^ In this study, we discovered that the glycan moiety of Globo H colocalized with A2AR at the cellular level and bound to the cytoplasmic domain of A2AR at the molecular level. Conversely, neither SSEA3 nor SSEA4, which differ from Globo H in their lack of terminal fucose, could bind to A2AR, aligning with their inability to influence Treg. These findings are reminiscent of the critical role of fucose moiety in binding GHCer to TRAX. Notably, this moiety also facilitates the direct interaction with the C‐terminus of A2AR.^[^ ^46^ ^]^ This is distinct from the previously reported nine lipid interaction sites for GM3, cholesterol, and PIP_2_ on the transmembrane helix of A2AR. While the C‐terminus of A2AR typically engages with proteins^[^ ^33^, ^47^, ^48^, ^49^, ^50^, ^51^ ^]^ in response to ligand‐induced structural changes, this study is the first to report its binding with a glycan. This interaction suggests that GHCer, a glycosphingolipid, regulates A2AR activity in a non‐canonical manner, independent of interactions between transmembrane domains of A2AR and lipid‐bilayer components. Understanding the interactions between the glycan moiety of GHCer, TRAX, and A2AR lays the groundwork for designing allosteric modulators that attenuate A2AR signaling. Interestingly, GHCer enhances the binding of TRAX to the shorter fragment E (aa 291–393) more than it does to the full‐length A2AR and fragment F (aa 291–412). Furthermore, fragment E shows a stronger interaction with GH‐Biotin compared to both full‐length A2AR and fragment F. It has been reported that the C‐terminus of A2AR plays a crucial role in the dimerization of A2AR,^[^ ^50^, ^51^, ^52^ ^]^ particularly at residue 394. Mutations at this site, as well as truncation of the C‐terminal tail (residues 394–412), have been shown to destabilize A2AR dimerization.^[^ ^50^ ^]^ Based on these findings, we hypothesize that the observed stronger interaction of fragment E with Globo H and TRAX, compared to full‐length A2AR or fragment F, may be related to the conformational changes in the dimeric forms of both full‐length A2AR and fragment F. However, this hypothesis requires further validation through additional experiments.

We observed significant upregulation of several inhibitory molecules, including LAG3, CTLA‐4, PD‐L1, IL‐35, CD39, CD73, and adenosine, in GHCer‐induced Treg cells. Among these, only adenosine can activate A2AR and promote the induction of Foxp3^+^ Treg cells,^[^ ^16^, ^53^ ^]^ Given that Treg cells are the primary adenosine provider in the tumor microenvironment (TME), the upregulation of ectonucleotidases, CD73, and CD39 on Treg cells by GHCer may further fuel adenosine generation, creating a positive feedback loop that fosters GHCer‐induced immunosuppression. In addition, adenosine has been shown to increase the surface expression of PD‐L1 and CTLA‐4 on Treg cells,^[^ ^54^ ^]^ potentially amplifying their suppressive functions.

In the tumor microenvironment, IL‐10 and IL‐35 were differentially expressed by Treg subpopulations^[^ ^55^, ^56^, ^57^, ^58^ and collaboratively promoted T cell exhaustion.^[^ ^59^ ^]^ Notably, we found that IL‐35, rather than IL‐10, is the predominant immunosuppressive cytokine in GHCer‐induced Treg cells. GHCer may drive Treg cells toward an IL‐35‐secreting subset by diminishing TCR signaling strength^[^ ^58^ ^]^ through the A2AR signaling pathway or LAG3 upregulation. We observed that GHCer blocks the phosphorylation of PLCγ1, an essential adaptor protein in the TCR signaling pathway, thereby reducing TCR signal strength. This aligns with A2AR signaling, which inhibits proximal TCR signaling through the activation of PKA and Src kinase, reducing recruitment of lymphocyte‐specific protein kinase (LCK) and reducing LCK recruitment to the CD3 of zeta‐chain–associated protein kinase‐70.^[^ ^60^ ^]^

Additionally, enhanced LAG3 expression in Treg cells induced by GHCer might further diminish TCR signal strength, as recent findings suggest that LAG3 also contributes to weakening. Recent findings indicate that LAG3 can dissociate the Lck protein from the TCR complex by lowering the pH in the immune synapse, thereby limiting T cell activation.^[^ ^61^ ^]^ Collectively, the adenosinergic pathway triggered by GHCer appears to influence the plasticity of Treg cells.

The advent of immune checkpoint inhibitors, such as anti‐CTLA‐4 and anti‐PD‐1/PD‐L1, has significantly transformed cancer therapy. However, not all patients benefit from such treatments. To enhance the anti‐cancer efficacy of immune checkpoint inhibitors, researchers are exploring combination therapies that target adenosinergic pathways. Given the crucial role of the adenosinergic pathway in tumor progression, strategies that block CD39, CD73, and/or A2AR may offer dual benefits, limiting tumor progression while restoring anti‐tumor immunity.^[^ ^62^, ^63^, ^64^, ^65^ ^]^ Several agents targeting these molecules are currently under clinical investigation.^[^ ^12^, ^26^ ^]^ For instance, the A2A receptor antagonist (SCH) combined with anti‐PD‐1 has shown a substantial reduction in both experimental and spontaneous metastases and has prolonged mouse survival compared to monotherapy.^[^ ^64^ ^]^

Furthermore, the combination of anti‐CD73 and anti‐PD‐1 has been shown to induce both antibody‐mediated responses and infiltration of cytotoxic T cells, leading to improved survival in a murine cancer model.^[^ ^54^ ^]^ Co‐inhibition of CD73 and A2AR signaling has enhanced anti‐tumor immune responses by promoting the expansion and infiltration of cytotoxic T cells, thereby reducing tumor growth and metastasis.^[^ ^66^ ^]^ So far, many clinical trials have been conducted to evaluate the efficacy of anti‐PD‐1 (PDR001, Atezolizumab, Durvalumab) in combination with various A2AR antagonists (PFB509, CPI‐444, AZD4635) or with a combination of an A2AR antagonist (CPI‐444) and a CD73 inhibitor (CPI‐006). Both monotherapies with A2AR antagonists and combination therapies with immune checkpoint blockade have shown favorable safety profiles and some clinical efficacy.^[^ ^67^, ^68^ ^]^ In this context, GHCer may represent a more promising target than A2AR and CD73. Targeting GHCer with immunotherapeutic agents or inhibitors could disrupt its interaction with TRAX and/or the C‐terminus of A2AR. This disruption may dampen A2AR activation, reduce CD39/73 expression and adenosine levels, and decrease the number of Treg cells and IL‐35 secretion.

Consequently, Globo H could serve as a cancer antigen and a target for immune checkpoint blockade. Unlike A2AR and CD39/73, GHCer has the distinct advantage of not being expressed in normal cells. Therefore, combining an anti‐Globo H agent with immune checkpoint inhibitors may offer a promising strategy, yielding potent and synergistic anti‐cancer efficacy.

## 4 Conclusion

In this study, we reveal GHCer as a novel immune checkpoint molecule that suppresses the activation of conventional T lymphocytes while promoting the differentiation and function of Treg cells. The GHCer‐induced immunosuppression is mediated through the activation of the A2AR/cAMP/PKA pathway, which involves the formation of complexes between TRAX and A2AR. Additionally, GHCer enhances the expression of the ectonucleotidases CD73 and CD39 in Tregs, thereby increasing the conversion of ATP to adenosine and establishing a positive feedback loop in A2AR signaling. These findings are further supported by a significant correlation between Treg cells in the tumor microenvironment and the expression of Globo H in clinical samples and animal tumor models. Globo H is expressed in over ten types of cancer and is associated with poor prognostic outcomes in several of them, generating considerable interest in the development of Globo H‐targeted therapeutics. Notably, the Globo H vaccine is currently undergoing a Phase III global clinical trial, making the identification of GHCer as an immune checkpoint molecule particularly timely. Our findings provide a strong scientific rationale for developing Globo H‐targeted therapeutics and lay the groundwork for designing small molecules that can disrupt the interactions between GHCer, A2AR, and TRAX. This represents a novel cancer‐specific strategy for A2AR blockade.

## 5 Experimental Section

### CD4^+^ T Cell Isolation, Activation, and Treg Differentiation

According to the manufacturer's instructions, splenocytes were collected aseptically from mice, and CD4^+^ T cells were enriched with a CD4^+^ T cell isolation kit (130‐117‐043, Miltenyi Biotech). Isolated cells, cultured in RPMI 1640 medium supplemented with 10% FBS, 4 mM L‐Glutamine, 50 U/ml penicillin, and 50 µg ml^−1^ streptomycin, were activated in plates coated with anti‐mouse CD3ε (1 µg ml^−1^) and anti‐mouse CD28 (0.5 µg ml^−1^) antibodies (100 339 and 102 115, BioLegend) up to 72 h or left unstimulated. Healthy donors were recruited from Chang Gung University with informed consent and approval from the ethics committee (Ethical approval number 5049/11). PBMCs were isolated from heparinized blood using Ficoll‐Paque Plus density gradient centrifugation (17‐144‐002, Cytiva), and CD4^+^ T cells were isolated by MACS separation (130‐097‐048, Miltenyi Biotech) according to the manufacturer's protocol. Cells were cultured in RPMI 1640 supplemented with 10% heat‐inactivated FBS, 2 mM l‐Glutamine, and 5 mM Glucose. CD4^+^ T cells (10^6^/ml) were cultured in 24‐well round‐bottom plates (Costar) and stimulated by anti‐CD3/CD28 Dynabeads (11131D, Thermo Fisher). In Treg differentiation experiments, CD4^+^ T cells were incubated with TGF‐β (5 ng ml^−1^) and IL‐2 (20 ng ml^−1^) (240‐B and BT‐002, R&D) during anti‐CD3/CD28‐stimulation for up to 6 days.

### Clinical Specimens

Tissue sections of human breast cancer and hepatocarcinoma were obtained from Chunghua Christian Hospital (Chunghua, Taiwan) and Tissue Bank of Linkou Chang Gung Memorial Hospital (Taoyuan, Taiwan) and were fully encoded to protect patient confidentiality. This study was approved by the Institutional Review Board of Human Subjects Research Ethics Committees of Chang Gung Memorial Hospital (IRB number: 201304321A3C108).

### Proliferation Assays

To measure cell proliferation, 5 × 10^5^ isolated CD4^+^ T cells were labeled with 1 µM carboxyfluorescein diacetate succinimidyl ester (CFSE; C34554, Life Technologies) for 10 min at 37 °C. Cells were washed to remove excess CFSE and then treated with 30 µM GHCer before being incubated with anti‐CD3ε/CD28 antibodies for 72 h. Proliferation was assessed by measuring CFSE fluorescence with flow cytometry analysis on a SA3800 Spectral Analyzer (SONY). Data were analyzed using FlowJo software.

### Adenosine Detection

T cells were incubated with GHCer in the presence or absence of FBS medium, and extracellular levels of ATP and adenosine were measured using an Adenosine assay kit (ab211094 and ab83355, Abcam), according to the manufacturer's instructions.

### ELISA Assays

IL‐10 and IL‐35 concentrations were measured in the supernatants of Treg differentiation cultures after 6 days, as described above, using specific ELISA kits (human: CSB‐E13126 h, CUSABIO; mouse: 440507, BioLegend) according to the manufacturer's instructions.

### cAMP Assays

According to the manufacturer's instructions, cAMP levels were measured with the LANCE cAMP assay kit (TRF0262, Revvity). 1 × 10^6^ anti‐CD3/CD28‐stimulated CD4^+^ T cells were incubated with 30 µM GHCer. At the indicated time point, supernatants were collected to determine cAMP levels.

### CREB Reporter Assay

Jurkat cells were transfected with the TransIT‐LT1 reagent (MIR 2304, Mirus) using the pGL4.29 (luc2P/CRE/Hygro) plasmid, which contains the Renilla gene for luciferase normalization. 24 h after transfection, cells were treated with NECA (119140, Sigma‐Aldrich), GHCer, or PBS. At 18 h post‐stimulation, the cell lysates were assayed for luciferase and Renilla activities using the Dual‐Luciferase assay system (E1910, Promega). Luciferase activities were normalized to Renilla activities.

### Immunohistochemistry

For Globo H staining, tissue sections were deparaffinized, followed by antigen retrieval using an autoclave at 121 °C for 5 min in AR‐10 solution (HK057‐5K‐GP, Biogenex). Slides were incubated with anti‐Globo H mAbVK9 antibody^[^ ^69^ ^]^ (provided by Dr. G. Ragupathi, Memorial Sloan‐Kettering Cancer Center, New York) (1:100 dilutions in antibody dilution buffer, Ventana Medical Systems, Inc.) overnight at 4 °C followed by polymer‐HRP IHC detection system (QD600‐GP, Biogenex). The slides were counter‐stained with hematoxylin and mounted. Human FOXP3 immunohistochemical staining was performed using the automated Ventana Benchmark XT with anti‐human FOXP3 (560 044, BD, 1:20, at room temperature) followed by a polymer‐HRP IHC detection system. Digital images were captured using an Aperio ScanScope XT Slide Scanner (Aperio Technologies, Vista, CA, USA) at 20 × magnification. The number of FOXP3 cells was evaluated using TissueStudio software (Definiens), which quantified 5–7 representative tumor regions to identify the percentage of positively stained FOXP3 cells among the total lymphocytes in the tumor region.

### Immunofluorescence Staining

CD4^+^ T cells and MDA‐MB‐231 cells were fixed with 4% PFA in PBS and permeabilized with 0.1% Triton X‐100. The cells were stained with GH‐biotin (1 mg ml^−1^) and an anti‐A2AR antibody (sc‐32261, Santa Cruz). GH‐biotin localization was detected by staining with streptavidin conjugated with Alexa Fluor 488 (405235, Biolegend); A2AR was detected by anti‐mouse IgG conjugated with Alexa Fluor 594 (405 326, Biolegend). The nucleus was stained with DAPI. The images were acquired using confocal microscopy (SP8, Leica).

### Coating of Luminex Beads with Antibody and Glycans

SSEA3‐amine, SSEA4‐amine, or Globo H‐amine^[^ ^70^ ^]^ (provided by Professor Chung‐Cheng, Lin) were coupled with beads (171506052, 171606006, and 171506071, BioRad) via a click reaction. Briefly, ≈2000 beads were washed with activation buffer twice and then activated with Sulfo‐N‐hydrosuccinimide (Sulfo‐NHS, 56485, Sigma‐Aldrich) and N‐[3‐dimethylaminopropyl]‐N’‐ethylcarbodiimide hydrochloride (EDC; 165334, Sigma‐Aldrich) by shaking for 20 min at room temperature. For other experiments, 1 µg anti‐His antibody (SAB4301134, Sigma‐Aldrich) was added to the activated beads and incubated for 2 h at room temperature. The coupling reaction's completion was evaluated by detecting the coupled antibodies or glycans using a phycoerythrin (PE)‐conjugated secondary antibody or anti‐glycan antibodies, respectively.

### Luminex‐Based Immunoassay

Assessment of protein/glycan interaction was carried out in a 96‐well plate. The A2AR fragments were suspended in 1 mL of PBS with 1% BSA and centrifuged at 14000 × g for 5 min; the supernatants were then placed in a new tube. The beads coated with the antibodies/glycans were vortexed for 30 s and then diluted in PBS/BSA to prepare the working solution at 5 × 10^5^ beads ml^−1^. To detect the TRAX/GHCer/A2AR complex, recombinant proteins, first antibodies (sc‐271632, Santa Cruz), and PE‐conjugated antibodies (405307 and 406421, BioLegend) were mixed and reacted at 37 °C for 60 min. Then, the working solution of antibodies/glycans‐coated beads was added and further incubated for 60 min at 37 °C. The recombinant A2AR fragments and anti‐His antibody were added to detect bindings with glycan‐conjugated beads. After incubation at 37 °C for 60 min, the median fluorescent intensity (MFI) of 200 beads was measured and analyzed using the Bio‐plex 200 system (Bio‐Rad).

### Thioacetamide (TAA)‐Induced Intrahepatic Cholangiocarcinoma in Rats

Adult male Sprague‐Dawley rats, averaging 318 ± 12 grams in weight, were obtained from the National Laboratory Animal Center (NLAC) in Taiwan and werehoused under specific‐pathogen‐free (SPF) conditions with unrestricted access to food and water. The animal studies were conducted following the guidelines for the Care and Use of Laboratory Animals and were approved by the Institutional Animal Care and Use Committee of Chang Gung Memorial Hospital (IACUC number 2018092502).

To induce intrahepatic cholangiocarcinoma, the rats were administered 300 mg L^−1^ of TAA (1.08170.0250, Merck)^[^ ^71^ ^]^ daily through their drinking water. After 24 weeks of TAA exposure, the rats were randomly assigned to receive either weekly intravenous injections of 300 µg of the Globo H antibody (mAbVK9 treated group, n = 5) or phosphate‐buffered saline (control group, n = 4) for two months. Tumor growth was monitored using PET scans, and the rats' body weights were recorded weekly throughout the treatment to detect any potential changes.

### Multiplex Immunohistochemical Staining

Rat tumor specimens were analyzed using multiplex immunohistochemical methods to detect and quantify Globo H and FOXP3, a marker for regulatory T cells. The slides were heated to 60 °C for 30 min, and paraffin was removed using Dewax (Leica Biosystems, Buffalo Grove, IL). Antigen retrieval was performed using ER2 solution (AR9640, Leica Biosystems) at 100 °C for 30 min. Then, the slides were washed and treated with a 3% hydrogen peroxide (H_2_O_2_) blocking solution to inhibit endogenous peroxidase activity. The primary antibody against Globo H (mAbVK9, 2.5 µg/ml; 14‐9700‐82, Invitrogen) was applied first, followed by incubation with the secondary antibody, Opal polymer HRP Ms + Rb (Akoya Biosystems, Marlborough, MA). Tyramide signal amplification (TSA‐dye 520, Opal 6‐Plex Manual Detection Kit, NEL871001KT, Akoya Biosystems) was then utilized, and the slides were heated again to strip the antibodies. This process was repeated for the antibody against FOXP3 (Ab215206, Abcam, 1:100 dilution) and its corresponding TSA dye (570). Finally, DAPI (1:1000 dilution, Perkin Elmer) was applied for nuclear staining. After the unbound DAPI was washed off, coverslips were mounted using Vectashield (Vector Laboratories, Burlingame, CA). Single‐color control slides were prepared using archival TAA‐induced rat cholangiocarcinoma samples to compare the staining results for Globo H and FOXP3 via standard immunohistochemistry methods. Digital images of the multiplex immunofluorescence staining were captured using a Leica DM6000 microscope with Leica Imaging Suite software. Exposure times for each fluorochrome were manually adjusted to minimize autofluorescence. Image analysis was conducted using Metamorph software (Molecular Devices, Inc.) to quantify the immune cells.

### Statistical Analysis

Statistical analysis was performed using Prism (GraphPad Software). All values are presented as means ± SD. Three independent experiments were conducted for each study, and representative results were presented. * p < 0.05, ** p < 0.01, *** p < 0.001. The p‐value was calculated using the student's t‐test or one‐way ANOVA.

## Conflict of Interest

Alice L. Yu is a consultant for OBI Pharma Inc. Other authors declare that they have no competing interests.

## Author Contributions

J.‐Y.C. and H.‐H.T. contributed equally to this work. Conceptualization: J.Y.C., J.T.H., A.L.Y. Methodology: J.Y.C., H.H.T., S.H.C., C.C.L., C.W.L. Investigation: Z.C.L., J.R.H., S.P.C., Y.H., F.Y.L. Visualization: J.Y.C., H.H.T. Supervision: J.T.H., J.Y., A.L.Y. Writing‐original draft: J.Y.C., H.H.T. Writing‐review & editing: J.Y.C, H.H.T., J.T.H., A.L.Y.

## Supporting information



Supporting Information

## Data Availability

The data that support the findings of this study are available from the corresponding author upon reasonable request.;

## References

[1] Y. C. Tsai , J. R. Huang , J. Y. Cheng , J. Lin , J. T. Hung , Y. Y. Wu , K. T. Yeh , A. L. Yu , J. Cancer Sci. Ther. 2013, 5, 264.

[2] B. A. Osborne , L. M. Minter , Nature reviews. Immunology 2007, 7, 64. 10.1038/nri1998 17170755

[3] C. N. Magee , N. Murakami , T. J. Borges , T. Shimizu , K. Safa , S. Ohori , S. Cai , A. Uffing , J. Azzi , W. Elyaman , L.‐M. Charbonnier , K. Liu , D. Toprak , G. Visner , T. A. Chatila , C. W. Siebel , N. Najafian , L. V. Riella , Circulation 2019, 140, 846. 31266349 10.1161/CIRCULATIONAHA.119.040563 PMC6722011

[4] I. T. Tran , A. R. Sandy , A. J. Carulli , C. Ebens , J. Chung , G. T. Shan , V. Radojcic , A. Friedman , T. Gridley , A. Shelton , P. Reddy , L. C. Samuelson , M. Yan , C. W. Siebel , I. Maillard , J. Clin. Invest. 2013, 123, 1590. 23454750 10.1172/JCI65477 PMC3613915

[5] C. Sorrentino , F. Hossain , P. C. Rodriguez , R. A. Sierra , A. Pannuti , S. Hatfield , B. A. Osborne , L. M. Minter , L. Miele , S. Morello , Frontiers in immunology 2019, 10, 162. 30792717 10.3389/fimmu.2019.00162 PMC6374329

[6] A. Ohta , M. Sitkovsky , Frontiers in immunology 2014, 5, 304. 25071765 10.3389/fimmu.2014.00304 PMC4091046

[7] M. Koshiba , D. L. Rosin , N. Hayashi , J. Linden , M. V. Sitkovsky , Mol. Pharmacol. 1999, 55, 614. 10051547

[8] M. R. Bono , D. Fernandez , F. Flores‐Santibanez , M. Rosemblatt , D. Sauma , FEBS Lett. 2015, 589, 3454. 26226423 10.1016/j.febslet.2015.07.027

[9] J. Blay , T. D. White , D. W. Hoskin , Cancer Res. 1997, 57, 2602. 9205063

[10] A. Ohta , Frontiers in immunology 2016, 7, 109. 27066002 10.3389/fimmu.2016.00109 PMC4809887

[11] A. Ohta , A. Ohta , M. Madasu , R. Kini , M. Subramanian , N. Goel , M. Sitkovsky , J. Immunol. 2009, 183, 5487. 19843934 10.4049/jimmunol.0901247

[12] D. Vijayan , A. Young , M. W. L. Teng , M. J. Smyth , Nature reviews. Cancer 2017, 17, 709. 29059149 10.1038/nrc.2017.86

[13] L. Antonioli , C. Blandizzi , P. Pacher , G. Hasko , Nature reviews. Cancer 2013, 13, 842. 24226193 10.1038/nrc3613

[14] P. E. Zarek , C.‐T. Huang , E. R. Lutz , J. Kowalski , M. R. Horton , J. Linden , C. G. Drake , J. D. Powell , Blood 2008, 111, 251. 17909080 10.1182/blood-2007-03-081646 PMC2200810

[15] M. Bettini , D. A. Vignali , Curr. Opin. Immunol. 2009, 21, 612. 19854631 10.1016/j.coi.2009.09.011 PMC2787714

[16] L. W. Collison , V. Chaturvedi , A. L. Henderson , P. R. Giacomin , C. Guy , J. Bankoti , D. Finkelstein , K. Forbes , C. J. Workman , S. A. Brown , J. E. Rehg , M. L. Jones , H.‐T. Ni , D. Artis , M. J. o. Turk , D. A. A. Vignali , Nat. Immunol. 2010, 11, 1093. 20953201 10.1038/ni.1952 PMC3008395

[17] A. Y. Wen , K. M. Sakamoto , L. S. Miller , J. Immunol. 2010, 185, 6413. 21084670 10.4049/jimmunol.1001829 PMC5519339

[18] C.‐N. Sun , H.‐C. Chuang , J.‐Y. Wang , S. i.‐Y. Chen , Y. a.‐Y. Cheng , C.‐F. Lee , Y. Chern , Developmental neurobiology 2010, 70, 604. 20506231 10.1002/dneu.20802

[19] J.‐Y. Cheng , S.‐H. Wang , J. Lin , Y. i.‐C. Tsai , J. Yu , J.‐C. Wu , J.‐T. Hung , J.‐J. Lin , Y.‐Y. Wu , K.‐T. u. Yeh , A. L. Yu , Cancer Res. 2014, 74, 6856. 25281721 10.1158/0008-5472.CAN-14-1651

[20] D. A. Vignali , L. W. Collison , C. J. Workman , Nature reviews. Immunology 2008, 8, 523. 10.1038/nri2343 PMC2665249 18566595

[21] C.‐T. Huang , C. J. Workman , D. Flies , X. Pan , A. L. Marson , G. Zhou , E. L. Hipkiss , S. Ravi , J. Kowalski , H. I. Levitsky , J. D. Powell , D. M. Pardoll , C. G. Drake , D. A. A. Vignali , Immunity 2004, 21, 503. 15485628 10.1016/j.immuni.2004.08.010

[22] N. Jain , H. Nguyen , C. Chambers , J. Kang , Proc. Natl. Acad. Sci. USA 2010, 107, 1524. 20080649 10.1073/pnas.0910341107 PMC2824392

[23] T. Kamada , Y. Togashi , C. Tay , D. Ha , A. Sasaki , Y. Nakamura , E. Sato , S. Fukuoka , Y. Tada , A. Tanaka , H. Morikawa , A. Kawazoe , T. Kinoshita , K. Shitara , S. Sakaguchi , H. Nishikawa , Proc. Nat. Acad. Sci. U. S. A. 2019, 116, 9999. 10.1073/pnas.1822001116 PMC6525547 31028147

[24] J. Gu , X. Ni , X. Pan , H. Lu , Y. Lu , J. Zhao , S. Guo Zheng , K. L. Hippen , X. Wang , L. Lu , Cell Mol. Immunol. 2017, 14, 521. 27374793 10.1038/cmi.2016.30 PMC5518817

[25] M. Mandapathil , B. Hilldorfer , M. J. Szczepanski , M. Czystowska , M. Szajnik , J. Ren , S. Lang , E. K. Jackson , E. Gorelik , T. L. Whiteside , The Journal of biological chemistry 2010, 285, 7176. 19858205 10.1074/jbc.M109.047423 PMC2844167

[26] R. D. Leone , L. A. Emens , J Immunother Cancer 2018, 6, 57. 29914571 10.1186/s40425-018-0360-8 PMC6006764

[27] M. Safford , S. Collins , M. A. Lutz , A. Allen , C.‐T. Huang , J. Kowalski , A. Blackford , M. R. Horton , C. Drake , R. H. Schwartz , J. D. Powell , Nat. Immunol. 2005, 6, 472. 15834410 10.1038/ni1193

[28] Y. Zheng , Y. Zha , G. Driessens , F. Locke , T. F. Gajewski , J. Exp. Med. 2012, 209, 2157. 23129747 10.1084/jem.20120342 PMC3501351

[29] B. Zhang , A. Jiao , M. Dai , D. L. Wiest , Y. Zhuang , J. Immunol. 2018, 201, 1452. 30012846 10.4049/jimmunol.1800106 PMC6103809

[30] Y. Yashiro‐Ohtani , Y. He , T. Ohtani , M. E. Jones , O. Shestova , L. Xu , T. C. Fang , M. Y. Chiang , A. M. Intlekofer , S. C. Blacklow , Y. Zhuang , W. S. Pear , Genes Dev. 2009, 23, 1665. 19605688 10.1101/gad.1793709 PMC2714710

[31] R. Kusko , J. Dreymann , J. Ross , Y. Cha , R. Escalante‐Chong , M. Garcia‐Miralles , L. J. Tan , M. E. Burczynski , B. Zeskind , D. Laifenfeld , M. Pouladi , M. Geva , I. Grossman , M. R. Hayden , Mol Neurodegener 2018, 13, 25. 29783994 10.1186/s13024-018-0259-3 PMC5963017

[32] S.‐H. Wang , J.‐Y. Cheng , H.‐H. Tsai , T.‐C. Lo , J.‐T. Hung , C.‐C. Lin , C.‐W. Lee , Y. i.‐H. Ho , H.‐H. Kuo , A. L. Yu , J. Yu , J Biomed Sci 2022, 29, 105. 36517806 10.1186/s12929-022-00889-w PMC9753400

[33] C.‐N. Sun , H.‐C. Cheng , J.‐L. Chou , S.‐Y. Lee , Y. a.‐W. Lin , H.‐L. Lai , H.‐M. Chen , Y. Chern , Mol. Pharmacol. 2006, 70, 454. 16617164 10.1124/mol.105.021261

[34] T. Chien , Y. u.‐T. Weng , S.‐Y. Chang , H.‐L. Lai , F.‐L. Chiu , H.‐C. Kuo , D. e.‐M. Chuang , Y. Chern , Mol. Psychiatry 2018, 23, 2375. 29298990 10.1038/s41380-017-0007-z PMC6294740

[35] T.‐H. Hung , J.‐T. Hung , C.‐E. n. Wu , Y. Huang , C.‐W. Lee , C.‐T. Yeh , Y. i.‐H. Chung , F.‐Y. Lo , L. i.‐C. Lai , J. K. Tung , J. Yu , C.‐N. Yeh , A. L. Yu , Hepatol Commun 2022, 6, 194. 34558839 10.1002/hep4.1800 PMC8710794

[36] S. Yang , Y. S. Cho , V. M. Chennathukuzhi , L. A. Underkoffler , K. Loomes , N. B. Hecht , The Journal of biological chemistry 2004, 279, 12605. 14711818 10.1074/jbc.M313133200

[37] Y.‐C. Wu , R. Williamson , Z. Li , A. Vicario , J. Xu , M. Kasai , Y. Chern , E. Tongiorgi , J. M. Baraban , J. Neurochem. 2011, 116, 1112. 21198640 10.1111/j.1471-4159.2010.07166.x PMC3050072

[38] Y. Liu , X. Ye , F. Jiang , C. Liang , D. Chen , J. Peng , L. N. Kinch , N. V. Grishin , Q. Liu , Science 2009, 325, 750. 19661431 10.1126/science.1176325 PMC2855623

[39] L. Li , W. Gu , C. Liang , Q. Liu , C. C. Mello , Y. i. Liu , Nat. Struct. Mol. Biol. 2012, 19, 824. 22773104 10.1038/nsmb.2337 PMC3414638

[40] J. Y. Wang , S. Y. Chen , C. N. Sun , T. Chien , Y. Chern , Oncogene 2016, 35, 1657. 26096928 10.1038/onc.2015.228

[41] K. Handa , S. I. Hakomori , Glycoconj J 2017, 34, 693. 27318475 10.1007/s10719-016-9684-0

[42] D. Russo , S. Parashuraman , G. D'Angelo , Int. J. Mol. Sci. 2016, 17, 1732. 27754465 10.3390/ijms17101732 PMC5085762

[43] S. H. Wang , T. J. Wu , C. W. Lee , J. Yu , Journal of Biomedical Science 2020, 27, 93. 32900381 10.1186/s12929-020-00684-5 PMC7487937

[44] Y. T. Tsai , Y. Itokazu , R. K. Yu , Neurochem. Res. 2016, 41, 107. 26498762 10.1007/s11064-015-1742-7 PMC4775412

[45] M. Sorice , P. Matarrese , A. Tinari , A. M. Giammarioli , T. Garofalo , V. Manganelli , L. Ciarlo , L. Gambardella , G. Maccari , M. Botta , R. Misasi , W. Malorni , FASEB J. 2009, 23, 3298. 19509307 10.1096/fj.08-128140

[46] K. D. Q. Nguyen , M. Vigers , E. Sefah , S. Seppälä , J. P. Hoover , N. S. Schonenbach , B. Mertz , M. A. O’Malley , S. Han , Elife. 2021, 10, e66662. 34269678 10.7554/eLife.66662 PMC8328514

[47] J. Burgueño , D. J. Blake , M. A. Benson , C. L. Tinsley , C. T. Esapa , E. I. Canela , P. Penela , J. Mallol , F. Mayor , C. Lluis , R. Franco , F. Ciruela , J. Biol. Chem. 2003, 278, 37545. 12837758 10.1074/jbc.M302809200

[48] A. S. Woods , D. Marcellino , S. N. Jackson , R. Franco , S. Ferré , L. F. Agnati , K. Fuxe , J. Proteome Res. 2008, 7, 3428. 18590318 10.1021/pr8001782 PMC2538563

[49] L. Canela , R. Luján , C. Lluís , J. Burgueño , J. Mallol , E. I. Canela , R. Franco , F. Ciruela , Mol. Cell. Neurosci. 2007, 36, 1. 17689978 10.1016/j.mcn.2007.05.007

[50] T. Milojevic , V. Reiterer , E. Stefan , V. M. Korkhov , M. M. Dorostkar , E. Ducza , E. Ogris , S. Boehm , M. Freissmuth , C. Nanoff , Mol. Pharmacol. 2006, 69, 1083. 16339847 10.1124/mol.105.015818

[51] G. Navarro , J. Hradsky , C. Lluís , V. Casadó , P. J. McCormick , M. R. Kreutz , M. Mikhaylova , Front Mol Neurosci 2012, 5, 53. 22529776 10.3389/fnmol.2012.00053 PMC3328853

[52] N. S. Schonenbach , M. D. Rieth , S. Han , M. A. O'Malley , FEBS Lett. 2016, 590, 3295. 27543907 10.1002/1873-3468.12367 PMC5039092

[53] A. Ohta , R. Kini , A. Ohta , M. Subramanian , M. Madasu , M. Sitkovsky , Front. Immunol. 2012, 3, 190. 22783261 10.3389/fimmu.2012.00190 PMC3389649

[54] B. Allard , S. Pommey , M. J. Smyth , J. Stagg , Clin. Cancer Res. 2013, 19, 5626. 23983257 10.1158/1078-0432.CCR-13-0545

[55] G. Niedobitek , D. Pazolt , M. Teichmann , O. Devergne , J Pathol 2002, 198, 310. 12375263 10.1002/path.1217

[56] M. A. Poleganov , M. Bachmann , J. Pfeilschifter , H. Muhl , Mol. Immunol. 2008, 45, 2869. 18336908 10.1016/j.molimm.2008.01.021

[57] R. Nishino , A. Takano , H. Oshita , N. Ishikawa , H. Akiyama , H. Ito , H. Nakayama , Y. Miyagi , E. Tsuchiya , N. Kohno , Y. Nakamura , Y. Daigo , Clin. Cancer Res. 2011, 17, 6272. 21849417 10.1158/1078-0432.CCR-11-0060

[58] J. C. Zeng , Z. Zhang , T. Y. Li , Y. F. Liang , H. M. Wang , J. J. Bao , J. A. Zhang , W. D. Wang , W. Y. Xiang , B. Kong , Z. Y. Wang , B. H. Wu , X. D. Chen , L. He , S. Zhang , C. Y. Wang , J. F. Xu , Int. J. Clin. Exp. Pathol. 2013, 6, 1806. 24040445 PMC3759487

[59] D. V. Sawant , H. Yano , M. Chikina , Q. Zhang , M. Liao , C. Liu , D. J. Callahan , Z. Sun , T. Sun , T. Tabib , A. Pennathur , D. B. Corry , J. D. Luketich , R. Lafyatis , W. Chen , A. C. Poholek , T. C. Bruno , C. J. Workman , D. A. A. Vignali , Nat. Immunol. 2019, 20, 724. 30936494 10.1038/s41590-019-0346-9 PMC6531353

[60] J. Linden , C. Cekic , Arterioscler Thromb Vasc Biol 2012, 32, 2097. 22772752 10.1161/ATVBAHA.111.226837 PMC4476649

[61] C. Guy , D. M. Mitrea , P. o.‐C. Chou , J. Temirov , K. M. Vignali , X. Liu , H. Zhang , R. Kriwacki , M. P. Bruchez , S. C. Watkins , C. J. Workman , D. A. A. Vignali , Nat. Immunol. 2022, 23, 757. 35437325 10.1038/s41590-022-01176-4 PMC9106921

[62] L. Antonioli , G. G. Yegutkin , P. Pacher , C. Blandizzi , G. Hasko , Trends Cancer 2016, 2, 95. 27014745 10.1016/j.trecan.2016.01.003 PMC4800751

[63] I. Perrot , H.‐A. Michaud , M. Giraudon‐Paoli , S. Augier , A. Docquier , L. Gros , R. Courtois , C. Déjou , D. Jecko , O. Becquart , H. Rispaud‐Blanc , L. Gauthier , B. Rossi , S. Chanteux , N. Gourdin , B. Amigues , A. Roussel , A. Bensussan , J.‐F. Eliaou , J. Bastid , F. Romagné , Y. Morel , E. Narni‐Mancinelli , E. Vivier , C. Paturel , N. Bonnefoy , Cell Rep. 2019, 27, 2411. 31116985 10.1016/j.celrep.2019.04.091

[64] P. A. Beavis , N. Milenkovski , M. A. Henderson , L. B. John , B. Allard , S. Loi , M. H. Kershaw , J. Stagg , P. K. Darcy , Cancer Immunol. Res. 2015, 3, 506. 25672397 10.1158/2326-6066.CIR-14-0211

[65] A. Ohta , E. Gorelik , S. J. Prasad , F. Ronchese , D. Lukashev , M. K. K. Wong , X. Huang , S. Caldwell , K. Liu , P. Smith , J.‐F. Chen , E. K. Jackson , S. Apasov , S. Abrams , M. Sitkovsky , Proc Natl Acad Sci U S A 2006, 103, 13132. 16916931 10.1073/pnas.0605251103 PMC1559765

[66] A. Young , S. F. Ngiow , D. S. Barkauskas , E. Sult , C. Hay , S. J. Blake , Q. Huang , J. Liu , K. Takeda , M. W. L. Teng , K. Sachsenmeier , M. J. Smyth , Cancer Cell 2016, 30, 391. 27622332 10.1016/j.ccell.2016.06.025

[67] K. Halpin‐Veszeleiova , S. M. Hatfield , Curr. Opin. Pharmacol. 2020, 53, 84. 32841869 10.1016/j.coph.2020.07.005

[68] R. C. Augustin , R. D. Leone , A. Naing , L. Fong , R. Bao , J. J. Luke , J Immunother Cancer 2022, 10, 004089. 10.1136/jitc-2021-004089 PMC8830302 35135866

[69] V. Kudryashov , G. Ragupathi , I. n. J. Kim , M. E. Breimer , S. J. Danishefsky , P. O. Livingston , K. O. Lloyd , Glycoconjugate J. 1998, 15, 243. 10.1023/a:1006992911709 9579801

[70] P.‐J. Li , S.‐Y. U. Huang , P.‐Y. Chiang , C.‐Y. O. Fan , L. I.‐J. Guo , D.‐Y. Wu , T. Angata , C.‐C. Lin , Angew Chem Int Ed Engl 2019, 58, 11273. 31140679 10.1002/anie.201903943

[71] C. N. Yeh , A. Maitra , K. F. Lee , Y. Y. Jan , M. F. Chen , Carcinogenesis 2004, 25, 631. 14656942 10.1093/carcin/bgh037

