# Supplementary material for: Tumor‐Associated Glycan Exploits Adenosine Receptor 2A Signaling to Facilitate Immune Evasion
Source: Adv Sci (Weinh). 2025 Jun 18;12(27):2416501. doi: 10.1002/advs.202416501 (PMC12279200; doi:10.1002/advs.202416501)
Supplement: Supplementary file 1 — Supporting Information [file ADVS-12-2416501-s001.docx]

Supporting Information for

**Tumor-Associated Glycan Exploits Adenosine Receptor 2A Signaling to Facilitate Immune Evasion**

Jing-Yan Cheng *et al.*

*Corresponding author. Email: a1yu@health.ucsd.edu&

johnyu@gate.sinica.edu.tw

**This PDF file includes:**

Supplementary Figure S1, S2, S3, and S4

Supplementary Methods

Supplementary Table 1

**Supplementary Figure S1**

***IL10***

***IL27P28***

***EBI3***

***IL35P35***

**Supplementary Figure S1. GHCer upregulates gene expression of *IL35* but not *IL10*.**

Quantitative PCR analyses of mRNA expression of *IL10*, *IL27P28*, *EBI3*, and *IL35P35* in CD4^+^ T- or Treg cells induced in the presence of PBS, 1 µM NECA, or 30 µM GHCer for 6 days. The mRNA levels were normalized to the level of *ACTB* and shown as a fold change of CD4^+^ T cell without activation.

Supplementary Figure S2

**PBS**

**NECA**

**GHCer**


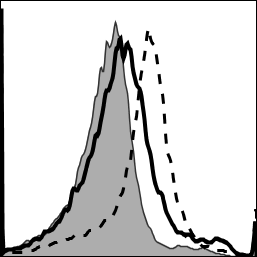


b


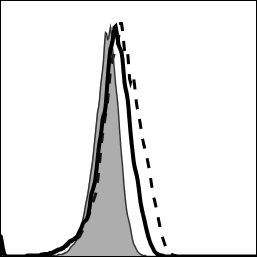

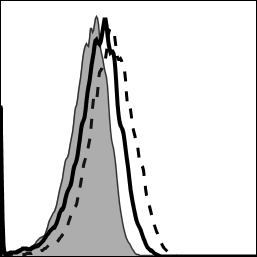


**LAG3**

**CTLA4**

**PDL1**

**

a

**Mouse IL-35**

**Conc. (pg/mL)**

**Supplementary Figure S2. GHCer induces inhibitory molecules and IL-35 expression in mouse Treg cells.** (**a**) GHCer increases expression of LAG3, CTLA4, and PDL1, and (**b**) secretion of IL-35 from mouse Treg induced in the presence of PBS, 1 μM NECA, or 30 μM GHCer, as determined by FACS and ELISA. Data represent three experiments, and values are expressed as means ± SD. Statistical significance was calculated using ANOVA with Tukey correction for multiple comparisons. ∗∗p < 0.01.

**Supplementary Figure S3**

**
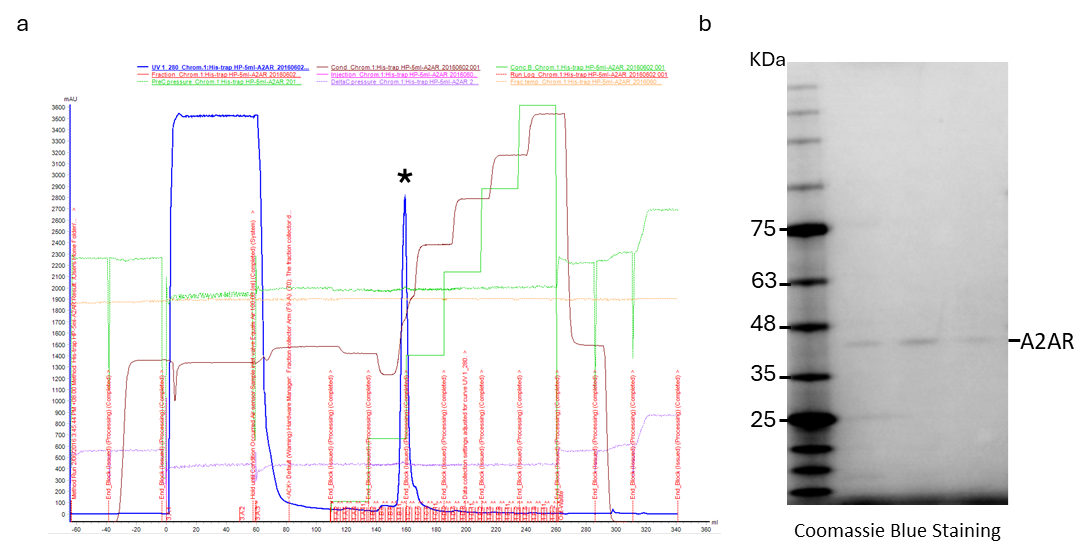
**

**Supplementary Figure S3. Purity Analysis of Purified A2AR Proteins.** (**a**) The blue line represents the UV absorbance at 280 nm, with an asterisk indicating a single peak that demonstrates the purity of the A2AR protein. (**b**) The presence of a single A2AR band in the SDS-PAGE confirms the purity and quality of the purified A2AR proteins.

**Supplementary Figure S4**

**
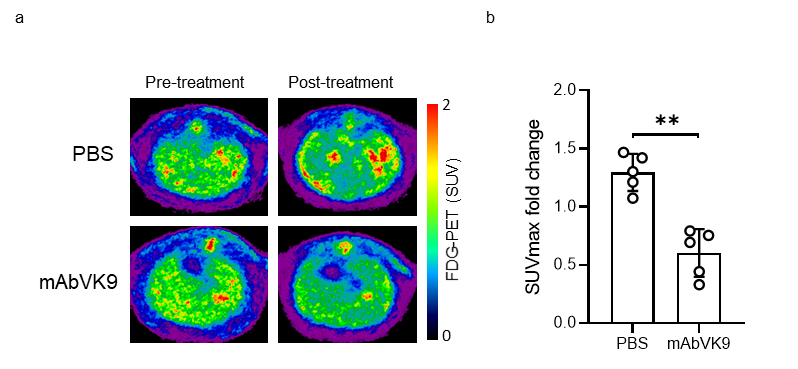
**

**Supplementary Figure S4. The anti-Globo H monoclonal antibody (mAbVK9) inhibits the progression of Globo H-expressing intrahepatic cholangiocarcinoma (ICC) in rats.** (**a**) Representative 18F-FDG PET images of rats bearing ICC treated at pre-treatment and after 2 months of treatment with either PBS or mAbVK9.(**b**) The bar graph illustrates the fold change in SUVmax (maximal standardized uptake value) from pre-treatment to post-treatment. mAbVK9 treatment significantly decreased SUVmax compared to the PBS group (p < 0.01).

**Supplementary Methods**

**Cells**

Jurkat and MDA-MB-231 cells were purchased from the Bioresource Collection and Research Center (Hsinchu, Taiwan). The Jurkat cell was cultured in RPMI 1640 medium (Corning); the MDA-MB-231 cell was cultured in Dulbecco's Modified Eagle's Medium (Corning) supplemented with 10% fetal bovine serum (FBS), 100 Unit/ml penicillin, and 100 μg/ml streptomycin at 37°C in a humidified 5% CO_2_ incubator.

**Mice**

Wild-type C57BL/6 mice (6-10 weeks old) purchased from NLAC, Taiwan and A2AR gene-deficient (A2AR^−/−^) C57BL/6-background mice (6-10 weeks old), kindly provided by Professor Yijuang Chern (Academia Sinica) were maintained at the animal facility of Chang Gung University and Chang Gung Memorial Hospital (IACUC number: CGU105-027 and 2021030301). Animal studies were conducted according to the guidelines for the Care and Use of Laboratory Animals and were approved by the Institutional Animal Care and Use Committee.

**Western Blot Analysis**

Jurkat cells were transfected with TransIT®-LT1 reagent (Mirus) using a plasmid containing the point mutations of the TRAX gene (Q219A, Q223A) with FLAG-tag in the C-terminal(*32*). Twenty-four hours after transfection, cells were lysed in cell lysis buffer (50 mM Tris-HCl, pH 7.4, 1% Triton X-100, 0.2% sodium deoxycholate, 0.2% SDS, 1 mM sodium EDTA) containing protease inhibitors (Roche). Protein concentrations were determined with Bio-Rad protein assay reagent. Equal proteins were electrophoretically separated in Nu-PAGEs and transferred to PVDF membranes (Immobilion-FL Transfer Membrane). Bovine serum albumin (5% BSA, Sigma) was used to block non-specific bindings. The following primary antibodies were used: anti-TRAX, anti-A2AR (both from Santa Cruz), and anti-FLAG (BioLegend). After incubation with appropriate secondary antibodies, immune-reactive protein bands were detected by enhanced chemiluminescence reagents (Amersham Pharmacia Biotech, UK) and analyzed by Typhoon (GE Healthcare). The optical densities of protein bands detected by Western blotting were calculated using ImageQuantTL (GE Healthcare).

**Quantitative Real-Time PCR**

According to the manufacturer's instructions, total RNAs were isolated using Tri-reagent (Invitrogen). Reverse transcription was performed using the cDNA Synthesis Kit (ABI). cDNAs were amplified by random primer; quantitative real-time PCR was performed using SYBR Green Supermix. Primer sequences were listed in **Supplementary Table 1**. Samples were triplicated in a 96-well Optical Reaction plate (Applied BioSystems). The PCR reaction conditions were 50 °C for 2 min, 95 °C for 5 min, 35 cycles of 95 °C for 30 s, 59 °C for 1 min, and 72 °C for 1 min, followed by a dissociation step. G. Results were analyzed using 7,500 fast system Software (Applied BioSystems).

**DNA construct and DNA transfection**

The mutation TRAX expression vector was subcloned from pPET-21d-TRAX ^Q219A, Q223A^ into the vector pLAS2w.Pbsd (Academia Sinica, Taipei, Taiwan) using the restriction enzymes NheI and EcoRI to obtain the plasmids for Flag-tagged TARX Q219A, Q223A expression. For lentiviral transduction, Jurkat cells and primary T cells were transduced with lentiviral particles at multiplicities of infection from 1 to 10. After 24 hours of infection, the culture media was replaced with RPMI 1640 with 10% FBS complete medium for another 48 hours. The lentiviral transfected cells were collected for further experiments.

**Protein expression and purification**

Human full-length TRAX, A2AR (A2AR-FL), and A2AR fragments (199-234, 291-393, and 291-412) were expressed or (33-42 and 101-120) synthesized (Kelowna, Taiwan) by N-terminal modifications with 9xHis. The cDNA fragments encoding the modified TRAX and A2AR fragments were produced by PCR using the primers list in supplementary table 1. The cloned genes were inserted into the pET-21d expression vectors. The expression plasmids were introduced into *E. coli* BL21-CodonPlus (DE3)-RIL (Stratagene). The recombinant proteins were induced with 1 mM IPTG at 15 °C overnight. The recombinant proteins were purified using Ni-NTA agarose as instructed by the manufacturer (Thermo Fisher).

**Reagents**

Cells were incubated with 1 μM A2AR agonist NECA (Sigma-Aldrich), 30 μM GHCer, A2AR antagonist SCH58261 (Sigma-Aldrich), and PKA antagonist H-89 (Sigma-Aldrich) alone or in combination, 30 min before activation with anti-CD3ε/CD28 antibodies.

**Flow cytometry**

Antibodies were purchased from BD Biosciences, BioLegend, or Invitrogen. For human cells, we used anti-CD4 (BioLegend), anti-CD25 (BioLegend), and anti-FOXP3 (BioLegend). Mouse cells were stained with anti-CD4 (BioLegend), anti-CD25 (BioLegend), and anti-FOXP3 (BioLegend). Jurkat cell was stained with anti-phospho-PLCγ1 (Ty783) (AbwizBio). The cells were incubated with antibodies for 20 min, washed, and fixed in Fix/Perm solution (BD Biosciences) for surface staining. The cells were stained intracellularly after being washed with Perm/Wash buffer for 30 min. Flow cytometry analysis on a SA3800 Spectral Analyzer (SONY). Data were analyzed using FlowJo v10.10 software.

**PET/CT Scan**

PET imaging was conducted using 18F-FDG as the radiotracer to assess treatment responses. Anesthesia was maintained with 3% isoflurane, and approximately 14.8 ± 1.5 MBq of 18F-FDG was administered via the tail vein. Static PET scans were obtained over 30 minutes, starting 90 minutes after the tracer injection, to capture the tumor's and surrounding tissues' metabolic activity(*66*). Micro-computed tomography (micro-CT) scans were performed subsequently for attenuation correction and anatomical co-registration. The Tera-Tomo 3D algorithm was utilized for image reconstruction, achieving a voxel size of 0.4 mm³, with corrections for the scatter and random events applied(*67*). Regions of interest (ROIs) for tumor and normal tissue were delineated using PMOD software (version 3.2; PMOD Technologies, Zurich, Switzerland). Tumor dimensions were determined from transverse images, focusing on areas of maximum diameter. The radiotracer uptake was quantified using standardized uptake values (SUV), specifically calculating the SUVmax for both the tumor and adjacent normal liver tissues. The tumor-to-liver radioactivity ratio (T/L ratio) was computed based on the SUVmax, following the guidelines set by the European Organization for Research and Treatment of Cancer to compare relative uptake. (*68*).

**Supplementary Table 1**.

**Primer sequences**

| **qPCR Primer** | |
| --- | --- |
| Human | |
| IL10-F | TGAGAACAGCTGCACCCACT |
| IL10-R | GGCAACCCAGGTAACCCTTA |
| TGFB-F | TGGAAACCCACAACGAAATC |
| TGFB-R | GGTTCAGGTACCGCTTCTC |
| IL27P28-F | GAGCAGCTCCCTGATGTTTC |
| IL27P28-R | AGCTGCATCCTCTCCATGTT |
| EBI3-F | GCAGCAGACGCCAACGT |
| EBI3-R | CCATGGAGAACAGCTGGACAT |
| IL35P35-F | CCTTCACCACTCCCAAAAC |
| IL35P35-R | TGTCTGGCCTTCTGGAGC |
| CD39-F | AGCAGCTGAAATATGCTGGC |
| CD39-R | GAGACAGTATCTGCCGAAGTCC |
| CD73-F | ATTGCAAAGTGGTTCAAAGTCA |
| CD73-R | ACACTTGGCCAGTAAAATAGGG |
| GADPH-F | ATTGCAAAGTGGTTCAAAGTCA |
| GADPH-R | ACCACCCTGTTGCTGTAGCCAA |
| CBLB-F | TGCCGATGCTAGACTTGGACGA |
| CBLB-R | TGATGTGACTGGTGAGTTCTGCC |
| ITCH-F | AGCGTAGTCAGCTTCAAGGAGC |
| ITCH-R | AGGTGGCAATGGACCAAGAGGA |
| ID3-F | CAGCTTAGCCAGGTGGAAATCC |
| ID3-R | GTCGTTGGAGATGACAAGTTCCG |
| EGR2-F | CCTTTGACCAGATGAACGGAGTG |
| EGR2-R | GAAGGTCTGGTTTCTAGGTGCAG |
| EGR3-F | GACTCGGTAGTCCATTACAATCAG |
| EGR3-R | AGTAGGTCACGGTCTTGTTGCC |
| **Mouse** | |
| Il2-F | GCGGCATGTTCTGGATTTGACTC |
| Il2-F | CCACCACAGTTGCTGACTCATC |
| Ifng-F | CAGCAACAGCAAGGCGAAAAAGG |
| Ifng-R | TTTCCGCTTCCTGAGGCTGGAT |
| Il4-F | GGATGAGAAGACAGGCTGGATAG |
| Il4-R | GCAGCTTCACATTATGCACCTCC |
| **Cloning** | |
| TRAX-F | AAGAATTCGAATGAGCAACAAAGAAGGATCAGG |
| TRAX-R | TTAAGCTTCTAGTGGTGGTGGTGGTGGTGGTGGTGGTGAG AAATGCCCTC TTCTTGATC |
| A2AR1-412-F | NNNNNGGATCCATGCCCATCATGGGCTCCTCG |
| A2AR1-412-R | NNNNNGAATTCCTAGTGGTGGTGGTGGTGGTGGTGGTGGTGGGACACTCCTGCTCCATCCTG |
| A2AR199-234-F | NNNNNGGATCCATGCGGATCTTCCTGGCGGCGCG |
| A2AR199-234-R | NNNNNGAATTCCTAGTGGTGGTGGTGGTGGTGGTGGTGGTGTGACTTGGCAGCATGGACCTC |
| A2AR291-393-F | NNNNNGGATCCATGCGTATCCGCGAGTTCCGCC |
| A2AR291-393-R | NNNNNGAATTCCCTAGTGGTGGTGGTGGTGGTGGTGGTGGTGACTCCCTTGAGCTCATGGC |
